# ﻿Discoveries of *Dothideomycetes (Fungi)* associated with pteridophytes in China

**DOI:** 10.3897/imafungus.16.167717

**Published:** 2025-12-17

**Authors:** Jing-Yi Zhang, Kevin D. Hyde, Ming-Fei Yang, Ya-Ru Sun, Xing-Juan Xiao, Ze-Bin Meng, Dan-Feng Bao, Yong-Zhong Lu

**Affiliations:** 1 School of Food and Pharmaceutical Engineering, Guizhou Institute of Technology, Guiyang 550025, China; 2 School of Public Health, Guiyang Healthcare Vocational University, Guiyang, Guizhou 550081, China; 3 Guizhou Key Laboratory of Agricultural Microbiology, Guizhou Academy of Agricultural Sciences, Guiyang 550009, China; 4 Center of Excellence in Fungal Research, Mae Fah Luang University, Chiang Rai 57100, Thailand; 5 School of Science, Mae Fah Luang University, Chiang Rai 57100, Thailand; 6 Guizhou Tea Seed Resource Utilization Engineering Research Center, Guizhou Education University, Guiyang 550018, China; 7 Engineering and Research Center for Southwest Bio-Pharmaceutical Resources of National Education Ministry of China, Guizhou University, Guiyang 550025, China

**Keywords:** *
Ascomycota
*, 13 new taxa, five new combinations, southwestern China, taxonomy

## Abstract

Pteridophytes are iconic symbols of the Earth’s biodiversity and harbor diverse fungal communities. In this study, an investigation of saprobic fungi associated with pteridophytes in China identified several fascinating taxonomic groups within *Dothideomycetes*. A polyphasic approach based on morphology, along with multi-gene phylogenetic analysis using combined LSU, *RPB2*, SSU, and *tef1-α* sequence data, revealed nine new collections representing five species in *Pleosporales* and five new collections representing two species in *Muyocopronales*. Consequently, six new genera (*Cyatheomyces*, *Microlepicola*, *Neoberkleasmium*, *Pseudopalawaniella*, *Synnematospora*, and *Xenopleopunctum*), six new species (*Cyatheomyces synnematosus*, *Microlepicola guizhouensis*, *Pseudopalawaniella woodwardiae*, *Synnematospora pronephrii*, *Xenopleopunctum guizhouense*, and *X. sporodochiale*), and five new combinations, viz., *Neoberkleasmium micronesiacum* (≡ *Berkleasmium micronesiacum*), *N. nigroapicale* (≡ *B. nigroapicale*), *Xenoberkleasmium crinisium* (≡ *B. crunisia*), *X. pandani* (≡ *B. pandani*), and *X. typhae* (≡ *B. typhae*), are proposed. Additionally, phylogenetic analysis reveals that four species of *Xenoberkleasmium* form a distinct lineage within *Pleosporales*, and they are evolving in a newly proposed family, *Xenoberkleasmiaceae*. Detailed morphological descriptions and a phylogenetic tree revealing the taxonomic placements of these new taxa are provided.

## ﻿Introduction

Fungi associated with pteridophytes (ferns and their allies) play crucial roles in plant colonization and offer unique insights into plant–fungal symbiosis, yet remain understudied (Remy et al. 1994; Guatimosim et al. 2016; Pressel et al. 2016). The most recent study conducted by Zhang et al. (2025) provides a comprehensive global exploration of fungi associated with pteridophytes and highlights that, based on a conservative estimate, over 92% of these fungi remain undiscovered.

*Pleosporales* was validly introduced by Barr (1987) and is the largest order within *Dothideomycetes*, *Ascomycota*, comprising approximately a quarter of all dothideomycetous species (Zhang et al. 2012a; Pem et al. 2024). *Pleosporales*, comprising approximately 93 families, represents a diverse and ecologically significant group of fungi that are globally distributed in terrestrial, marine, and freshwater habitats (Zhang et al. 2009, 2012a; Hongsanan et al. 2020a; Pem et al. 2024). These species include epiphytes, saprobes, endophytes, parasites, pathogens, lichens, and hyperparasites of fungi, insects, or mammals, with a notable prominence on plant hosts (Zhang et al. 2009, 2012a; Hyde et al. 2013; Li et al. 2023; Pem et al. 2024).

*Muyocopronales* was introduced by Mapook et al. (2016b) to accommodate a single family, *Muyocopronaceae*. Phylogenetically, *Palawaniaceae* was introduced in the *Dothideomycetes* family incertae sedis (Mapook et al. 2016a; Hongsanan et al. 2020b) and was later presumed to belong to the *Muyocopronales* based on morphological similarity with *Muyocopronales* and the MCC tree (Mapook et al. 2016a, 2020a). However, subsequent studies did not support this placement (Hongsanan et al. 2020b; Mapook and Hyde 2023). Currently, *Muyocopronaceae* is the only family included in *Muyocopronales* (Senwanna et al. 2021; Xu et al. 2024). These species have a widespread distribution, commonly occurring as pathogens or saprobes on various plant substrates (Tibpromma et al. 2016; Crous et al. 2018a; Hernández-Restrepo et al. 2019; Senwanna et al. 2019a), sometimes occurring on soil (Madrid et al. 2012; Hernández-Restrepo et al. 2019), while some are pathogens infecting animals and humans (Hernández-Restrepo et al. 2019).

In this study, we investigated the diversity of microfungi on decayed parts of ferns in China, obtaining 14 fresh collections representing seven *Dothideomycetes* species. Morphological and phylogenetic analyses provide further evidence for the classification of these species. This study is an extension of the research conducted by Zhang et al. (2025), focusing on fungal taxonomy with the aims of 1) investigating the diversity of saprobic fungi associated with ferns, drawing attention to this fungal group, and 2) describing novel taxa based on both morphological characteristics and phylogenetic evidence, enriching the fungal resources related to ferns.

## ﻿Methods

### ﻿Collections, morphology, and isolation

Decayed rachides, or petioles, of ferns were collected from terrestrial habitats in China. Specimens were examined to determine the presence of fungi on the host substrate using a stereomicroscope (SMZ168-BL, Motic, China), as well as their macromorphological characters. Micro-morphological features of fungi were observed and photographed using an ECLIPSE Ni-U compound microscope (Nikon, Japan) fitted with an EOS 90D digital camera (Canon, Japan). Measurements were made by Tarosoft (R) Image Frame Work v.0.9.7. Photo-plates were processed and combined with Adobe Photoshop CC 2019 (Adobe Systems, USA).

Single-spore isolations were made onto water agar (WA; 16 g/L distilled water) or potato dextrose agar (PDA; 39 g/L distilled water, Difco potato dextrose), and germinated spores were transferred onto PDA following the method in Senanayake et al. (2020). Pure culture plates were incubated at 26 °C for 3–8 weeks. Dried specimens were deposited in the Herbarium of Kunming Institute of Botany, Academia Sinica (HKAS), Chinese Academy of Sciences, Kunming, China, and the Herbarium of Guizhou Academy of Agricultural Sciences (GZAAS), Guiyang, China. Pure living cultures were deposited in the Kunming Institute of Botany Culture Collection (KUNCC) and Guizhou Culture Collection, China (GZCC). Names of the novel taxa were registered in Index Fungorum (http://www.indexfungorum.org).

### ﻿DNA extraction, amplification, sequencing

Fungal genomic DNA was extracted from fresh mycelia grown on PDA, following the manufacturer’s instructions as described in the Biospin Fungus Genomic DNA Extraction Kit (Biospin Fungus Genomic DNA Extraction Kit, BioFlux®, Shanghai, China). Polymerase chain reactions (PCR) were carried out using the following primers: NS1 and NS4 (White et al. 1990), ITS5 and ITS4 (White et al. 1990), LR0R and LR5 (Vilgalys and Hester 1990), fRPB2-5F and fRPB2-7cR (Liu et al. 1999), and EF1-983F and EF1-2218R (Rehner and Buckley 2005). These primers were used to amplify the 18S subunit rDNA (SSU), the internal transcribed spacer (ITS), the large subunit of ribosomal DNA (LSU), the RNA polymerase II subunit 2 (*RPB2*), and the translation elongation factor 1 (*tef1-α*) gene regions, respectively. The amplification conditions were based on the protocol described by Zhang et al. (2021). The quality of the PCR products was checked on 1% agarose gel stained with ethidium bromide. Successful PCR products were sent to Sangon Biotech (Shanghai, China) for purification and sequencing. The sequences generated in this study have been deposited in NCBI GenBank.

### ﻿Sequence alignments and phylogenetic analysis

Forward and reverse sequence reads were assembled using SeqMan v. 7.0.0 (DNASTAR, Madison, WI). Consensus sequences were subjected to a BLASTn search in NCBI GenBank (https://blast.ncbi.nlm.nih.gov/Blast.cgi) to select taxa for subsequent phylogenetic analyses. Consequently, representative sequence data of each family from *Pleosporales*, *Muyocopronales*, and several major related lineages used for phylogenetic analyses in *Dothideomycetes* were selected based on BLASTn searches, as well as recent publications (Table 1). Sequences newly generated in this study were deposited in GenBank. Sequence alignments for different gene loci were performed using the online multiple alignment program MAFFT version 7.2 (https://mafft.cbrc.jp/alignment/server/; Katoh et al. 2019). Trimal v. 1.2 was used to remove ambiguously aligned regions and uninformative positions with the “gt = 0.6” option (Capella-Gutiérrez et al. 2009). The obtained alignment was deposited in Figshare (https://figshare.com/; Suppl. material 1). Sequences of each locus were combined to form a concatenated supermatrix using SequenceMatrix 1.7.8 (Vaidya et al. 2011) and analyzed with maximum likelihood (ML) and Bayesian inference (BI).

**Table 1.** Taxa used in this study and their GenBank accession numbers.

Taxa	Strain No.	GenBank Accession Numbers				References
		LSU	*RPB2*	SSU	*tef*1-*α*	
*Acrocalymma medicaginis*	CPC 24340	KP170713	N/A	N/A	N/A	Trakunyingcharoen et al. (2014)
*Acrocalymma pterocarpi*	MFLUCC 17-0926	MK347949	MK434897	MK347840	MK360040	Jayasiri et al. (2019)
*Aigialus parvus*	BCC 18403	GU479778	GU479817	GU479744	GU479842	Suetrong et al. (2009)
*Aigialus rhizophorae*	BCC 33572	GU479780	GU479819	GU479745	GU479844	Suetrong et al. (2009)
*Alternaria alternata*	CBS 916.96	DQ678082	DQ677980	DQ678031	DQ677927	Schoch et al. (2006a)
*Amniculicola aquatica*	MFLUCC 16-1123	MK106096	N/A	N/A	MK109800	Hyde et al. (2019)
*Amniculicola asexualis*	GZCC 20-0482	OP377926	OP473094	OP378011	OP473006	Yang et al. (2023)
*Amorocoelophoma cassiae*	MFLUCC 17-2283	MK347956	MK434894	NG_065775	MK360041	Jayasiri et al. (2019)
*Angustimassarina lonicerae*	MFLUCC 15-0087	KY496724	N/A	N/A	N/A	Tibpromma et al. (2017)
*Anteaglonium parvulum*	SMH5223	GQ221909	N/A	N/A	GQ221918	Mugambi and Huhndorf (2009a)
*Aposphaeria corallinolutea*	MFLU 15-2752	KY554197	KY554207	KY554200	KY554205	Tibpromma et al. (2017)
*Aquasubmersa japonica*	HHUF 30469	NG_057138	LC194421	NG_062426	LC194384	Ariyawansa et al. (2015a); Hashimoto et al. (2017a)
*Aquasubmersa mircensis*	MFLUCC 11-0401	NG_042699	N/A	NG_061141	N/A	Zhang et al. (2012b)
*Arxiella longispora*	SGSF 303	MW519910	MW717995	N/A	MW883564	Xu et al. (2023a)
*Ascocylindrica marina*	MD6011	KT252905	N/A	KT252907	N/A	Ariyawansa et al. (2015a)
*Ascocylindrica marina*	MF416	MK007123	N/A	MK007124	N/A	Ariyawansa et al. (2015a)
*Astragalicola vasilyevae*	MFLUCC 17-0832	MG828986	MG829248	MG829098	MG829193	Wanasinghe et al. (2018)
*Astrosphaeriella fusispora*	MFLUCC 10-0555	KT955462	KT955413	KT955443	KT955425	Phookamsak et al. (2015)
*Austropleospora ochracea*	GZCC 19-0430	MW133817	N/A	MW134597	OP473010	Yang et al. (2023)
*Bahusandhika indica*	GUFCC 18001	KF460274	N/A	N/A	N/A	Pratibha et al. (2014)
*Bambusicola bambusae*	MFLUCC 11-0614	JX442035	KP761718	JX442039	KP761722	Dai et al. (2012, 2015)
*Bambusicola massarinia*	MFLUCC 11-0389	NG_058658	KP761716	NG_061198	KP761725	Dai et al. (2012)
*Berkleasmium ariense*	NFCCI 4026	KY039165	N/A	N/A	N/A	Tibpromma et al. (2017)
*Berkleasmium crunisia*	BCC 17023	DQ280271	N/A	N/A	N/A	Pinnoi et al. (2007)
*Berkleasmium micronesiacum*	BCC 8141	DQ280272	N/A	DQ280268	N/A	Pinnoi et al. (2007)
*Berkleasmium nigroapicale*	BCC 8220	DQ280273	N/A	DQ280269	N/A	Pinnoi et al. (2007)
*Berkleasmium typhae*	BCC 12536	DQ280275	N/A	N/A	N/A	Pinnoi et al. (2007)
*Botryosphaeria dothidea*	CMW 8000	KF766319	DQ677944	KF766233	DQ767637	Schoch et al. (2006a)
*Brevicollum versicolor*	HHUF 30591	NG_058716	LC271250	NG_065124	LC271246	Tanaka et al. (2017)
*Camarosporidiella caraganicola*	MFLUCCC 14-0605	KP711381	N/A	KP711382	N/A	Liu et al. (2015)
*Camarosporium quaternatum*	CPC 31081	NG_064442	N/A	KY929123	KY929201	Crous and Groenewald (2017)
*Camarosporomyces flavigenus*	CBS 314.80	GU238076	N/A	NG_061093	N/A	Aveskamp et al. (2010)
*Capnodium coffeae*	CBS 147.52	DQ247800	KT216519	DQ247808	DQ471089	Schoch et al. (2006b); Spatafora et al. (2006); Ismail et al. (2016);
*Capnodium salicinum*	CBS 131.34	DQ678050	KT216553	DQ677997	DQ677889	Schoch et al. (2006a)
*Clematidis italica*	MFLUCC 15-0084	KU842381	N/A	NG_061236	N/A	Li et al. (2016)
*Clypeoloculus akitaensis*	KT 788	AB807543	N/A	AB797253	AB808519	Tanaka et al. (2015)
*Coniothyrium palmarum*	CBS 400.71	JX681084	KT389592	EU754054	DQ677903	Schoch et al. (2006a)
*Corynespora cassiicola*	CBS 100822	GU301808	GU371742	GU296144	GU349052	Schoch et al. (2009)
*Corynespora torulosa*	CPC 15989	KF777207	N/A	N/A	N/A	Crous et al. (2013)
*Crassiperidium octosporum*	MAFF 242971	NG_066389	LC373132	NG_065689	LC373120	Matsumura et al. (2018)
*Cryptocoryneum japonicum*	HHUF 30482	NG_059035	LC194438	NG_065118	LC096144	Hashimoto et al. (2017a)
*Cryptocoryneum pseudorilstonei*	CBS 113641	NG_059036	LC194446	LC194322	LC096152	Hashimoto et al. (2017a)
*Cucurbitaria berberidis*	MFLUCC 11-0387	KC506796	N/A	KC506800	N/A	Doilom et al. (2013)
*Cyatheomyces synnematosus*	KUNCC 23-13865	PV862390	PV948862	N/A	PV948875	This study
*Cyatheomyces synnematosus*	GZCC 23-0670	PV862391	PV948863	N/A	PV948876	This study
*Cyatheomyces synnematosus*	KUNCC 23-14156	PV862392	PV948864	N/A	PV948877	This study
*Cyatheomyces synnematosus*	KUNCC 23-14159	PV862393	PV948865	N/A	PV948878	This study
*Cyclothyriella rubronotata*	CBS 141486	KX650544	KX650574	NG_061252	KX650519	Jaklitsch et al. (2016)
*Cyclothyriella rubronotata*	CPC 27604	MH107933	N/A	N/A	N/A	Crous et al. (2018b)
*Dacampia engeliana*	Hafellner 72868	KT383791	N/A	N/A	N/A	Ertz et al. (2015)
*Dacampia hookeri*	Hafellner 73897	KT383792	N/A	N/A	N/A	Ertz et al. (2015)
*Delitschia chaetomioides*	SMH 3253.2	GU390656	N/A	N/A	GU327753	Mugambi and Huhndorf (2009b)
*Delitschia winteri*	AFTOL-ID 1599	DQ678077	DQ677975	DQ678026	DQ677922	Schoch et al. (2006a)
*Dendrographa decolorans*	Ertz 5003 (BR)	AY548815	DQ883715	AY548809	DQ883725	Spatafora et al. (2006)
*Dictyocheirospora bannica*	KH 332	AB807513	N/A	AB797223	AB808489	Tanaka et al. (2015)
*Didymella exigua*	CBS 183.55	MH868977	GU371764	N/A	N/A	Schoch et al. (2009); Vu et al. (2019)
*Didymella rumicicola*	CBS 683.79	MH873007	KT389622	N/A	N/A	Vu et al. (2019)
*Didymosphaeria rubi-ulmifolii*	MFLUCC 14-0023	KJ436586	N/A	KJ436588	N/A	Ariyawansa et al. (2014)
*Digitodesmium chishuiense*	GZCC 20-0510	OP377907	OP473082	OP377993	OP472990	Yang et al. (2023)
*Dothidotthia aceris*	MFLUCC 16-1183	MK751816	N/A	MK751761	N/A	Senwanna et al. (2019b)
*Equiseticola fusispora*	MFLUCC 14-0522	NG_059249	N/A	NG_061238	MG520895	Abd-Elsalam et al. (2016)
*Fuscostagonospora cytisi*	MFLUCC 16-0622	KY770978	N/A	KY770977	KY770979	Hyde et al. (2017)
*Fuscostagonospora sasae*	HHUF 29106	AB807548	N/A	AB797258	AB808524	Tanaka et al. (2015)
*Fusculina eucalypti*	CBS 145083	MK047499	N/A	N/A	N/A	Crous et al. (2018a)
*Gordonomyces mucovaginatus*	CBS 127273	NG_057941	N/A	N/A	N/A	Crous et al. (2011a)
*Halobyssothecium aquifusiforme*	GZCC 20-0481	OP377925	OP473093	OP378010	OP473005	Yang et al. (2023)
*Halojulella avicenniae*	BCC 20173	GU371822	GU371786	GU371830	GU371815	Schoch et al. (2009)
*Halojulella avicenniae*	JK 5326A	GU479790	N/A	GU479756	N/A	Suetrong et al. (2009)
*Halotthia posidoniae*	BBH 22481	GU479786	N/A	GU479752	N/A	Suetrong et al. (2009)
*Helicosporium liuzhouense*	GZCC 22-2014	OQ981402	OQ980474	N/A	OQ980476	Xiao et al. (2023)
*Helminthosporium velutinum*	L131	KY984352	KY984413	KY984432	KY984463	Voglmayr and Jaklitsch (2017)
*Hermatomyces iriomotensis*	HHUF 30518	LC194367	LC194449	LC194325	LC194394	Hashimoto et al. (2017a)
*Hermatomyces jinghaensi*s	HKAS 112167	MW989519	N/A	N/A	MZ042642	Ren et al. (2021)
*Hermatomyces turbinatus*	MFLUCC 21-0038	MW989518	MZ042638	N/A	MZ042641	Ren et al. (2021)
*Hyphodiscosia jaipurensis*	MFLU 23-0472	PP112042	N/A	PP101307	N/A	Xu et al. (2024)
*Hyphodiscosia jaipurensis*	MFLUCC 23-0302	PP112041	N/A	PP101306	N/A	Xu et al. (2024)
*Hypsostroma caimitalense*	GKM1165	GU385180	N/A	N/A	N/A	Mugambi and Huhndorf (2009b)
*Hypsostroma saxicola*	SMH 5005	GU385181	N/A	N/A	N/A	Mugambi and Huhndorf (2009b)
*Hysterium angustatum*	CBS 123334	FJ161207	FJ161129	FJ161167	FJ161111	Boehm et al. (2009)
*Hysterobrevium smilacis*	CBS 114601	FJ161174	FJ161114	FJ161135	FJ161091	Boehm et al. (2009)
*Latorua caligans*	CBS 576.65	NG_058180	N/A	N/A	N/A	Crous et al. (2015)
*Latorua grootfonteinensis*	CBS 369.72	NG_058181	N/A	N/A	N/A	Crous et al. (2015)
*Lecanactis abietina*	Ertz 5068 (BR)	AY548812	AH013900	AY548805	N/A	Lutzoni et al. (2004)
*Lentimurispora urniformis*	MFLUCC 18-0497	MH179144	N/A	MH179160	MH188055	Liu et al. (2018a)
*Lentithecium pseudoclioninum*	HHUF 29055	NG_059392	N/A	NG_064847	AB808521	Tanaka et al. (2015)
*Leptodiscella africana*	CBS 400.65	MH870275	MK492711	N/A	MK495955	Hernández-Restrepo et al. (2019)
*Leptodiscella sexualis*	MFLU 19-2783	MW293930	N/A	N/A	N/A	Tennakoon et al. (2021)
*Leptosphaeria cichorium*	MFLUCC 14-1063	KT454712	N/A	KT454728	N/A	Ariyawansa et al. (2015b)
*Leptoxyphium fumago*	CBS 123.26	GU301831	GU371741	GU296161	GU349051	Schoch et al. (2009)
*Libertasomyces myopori*	CPC 27354	NG_058241	N/A	N/A	N/A	Crous et al. (2016)
*Ligninsphaeria jonesii*	GZCC 15-0080	KU221038	N/A	N/A	N/A	Zhang et al. (2016a)
*Ligninsphaeria jonesii*	MFLUCC 15-0641	NG_059642	N/A	N/A	N/A	Zhang et al. (2016a)
*Lignosphaeria thailandica*	MFLUCC 11-0376	KP888645	N/A	N/A	N/A	Thambugala et al. (2015)
*Lindgomyces cigarosporus*	G619	KX655804	N/A	KX655805	N/A	Raja et al. (2017)
*Lindgomyces ingoldianus*	ATCC 200398	AB521736	N/A	NG_016531	N/A	Hirayama et al. (2010)
*Longipedicellata aptrootii*	MFLU 10-0297	KU238894	KU238891	KU238895	KU238892	Zhang et al. (2016b)
*Longipedicellata aquatica*	MFLUCC 15-0630	OP377961	OP473115	OP378038	OP473053	Yang et al. (2023)
*Lophiostoma macrostomum*	KT508	AB619010	JN993491	AB618691	LC001751	Hirayama and Tanaka (2011); Schoch et al. (2012); Thambugala et al. (2015)
*Lophiotrema fallopiae*	MAFF 245612	LC149915	LC194459	LC149911	LC194404	Hyde et al. (2016)
*Lophiotrema nucula*	CBS 627.86	GU301837	GU371792	GU296167	GU349073	Schoch et al. (2009)
*Macrodiplodiopsis desmazieri*	CBS 140062	NG_058182	N/A	N/A	N/A	Crous et al. (2015)
*Macrodiplodiopsis desmazieri*	CBS 222.37	KR909316	KR909322	KR909318	KR909319	Ahmed et al. (2015)
*Magnicamarosporium iriomotense*	HHUF 30125	NG_059389	N/A	AB797219	AB808485	Tanaka et al. (2015)
*Massaria anomia*	CBS 591.78	GU301839	GU371769	GU296169	N/A	Schoch et al. (2009)
*Massaria inquinans*	WU 30527	HQ599402	HQ599460	HQ599444	HQ599342	Voglmayr et al. (2011)
*Mauritiana rhizophorae*	BCC 28866	GU371824	N/A	GU371832	GU371817	Schoch et al. (2009)
*Melanomma japonicum*	MAFF 239634	NG_060360	LC203395	NG_065122	LC203367	Hashimoto et al. (2017b)
*Melanomma pulvis-pyrius*	CBS 124080	MH874873	GU456350	GU456302	GU456265	Zhang et al. (2009)
*Microlepicola guizhouensis*	KUNCC 23-14007	PV862399	PV948870	PV862385	PV948884	This study
*Morosphaeria muthupetensis*	PUFD87	MF614796	N/A	MF614797	MF614798	Devadatha et al. (2018)
*Morosphaeria velatispora*	KH 221	AB807556	N/A	AB797266	AB808532	Tanaka et al. (2015)
*Muyocopron chromolaenae*	MFLUCC 17-1513	NG_068700	MT136761	NG_070150	MT136756	Mapook et al. (2020a)
*Muyocopron dipterocarpi*	MFLUCC 14-1103	KU726966	KY225779	KU726969	MT136754	Mapook et al. (2016b)
*Mycoleptodiscus endophyticus*	MFLUCC 17-0545	MG646946	N/A	NG_065724	MG646985	Tibpromma et al. (2018b)
*Mycoleptodiscus suttonii*	CBS 276.72	MK487728	MK492732	N/A	MK495974	Hernández-Restrepo et al. (2019)
*Mycoleptodiscus terrestris*	IMI 159038	MK487731	MK492735	N/A	MK495977	Hernández-Restrepo et al. (2019)
*Neoastrosphaeriella krabiensis*	MFLUCC 11-0025	JN846729	N/A	JN846739	N/A	Liu et al. (2011)
*Neocamarographium carpini*	CBS 128781	JQ044450	N/A	N/A	N/A	Crous et al. (2011b)
*Neocamarosporium goegapense*	CBS 138008	KJ869220	N/A	N/A	N/A	Crous et al. (2014)
*Neocamarosporium phragmitis*	MFLUCC 17-0756	NG_070431	N/A	NG_065736	MG844351	Hyde et al. (2018)
*Neocochlearomyces chromolaenae*	BCC 68250	NG_066431	N/A	NG_065766	MK047573	Crous et al. (2018a)
*Neocochlearomyces chromolaenae*	BCC 68252	MK047516	N/A	MK047554	MK047575	Crous et al. (2018a)
*Neodeightonia palmicola*	MFLUCC 10-0822	HQ199222	N/A	HQ199223	N/A	Liu et al. (2010)
*Neohendersonia kickxii*	CBS 112403	NG_058264	N/A	N/A	N/A	Giraldo et al. (2017)
*Neokalmusia aquibrunnea*	GZCC 17-0045	OP377920	N/A	OP378005	OP473000	Yang et al. (2023)
*Neomassaria fabacearum*	MFLUCC 16-1875	KX524145	N/A	NG_061245	KX524149	Hyde et al. (2016)
*Neomassaria formosana*	NTUCC 17-007	MH714756	MH714765	MH714759	MH714762	Ariyawansa et al. (2018)
*Neomassarina chromolaenae*	MFLUCC 17-1480	NG_068715	MT235822	NG_070168	MT235785	Mapook et al. (2020a)
*Neomassarina thailandica*	MFLUCC 17-1432	MT214467	MT235823	MT214420	MT235786	Mapook et al. (2020a)
*Neomassariosphaeria aquimucosa*	GZCC 19-0500	MW133803	N/A	MW134591	OP473014	Yang et al. (2023)
*Neomycoleptodiscus alishanense*	MFLUCC 19-0390	ON024150	N/A	N/A	N/A	Liu et al. (2024b)
*Neomycoleptodiscus venezuelense*	CBS 100519	NG_066340	MK492736	N/A	MK495978	Hernández-Restrepo et al. (2019)
*Neooccultibambusa thailandensis*	MFLUCC 16-0274	NG_068827	MH412758	MH260348	MH412780	Tibpromma et al. (2018a)
*Neopaucispora rosaecae*	MFLUCC 17-0807	MG829033	N/A	NG_061293	MG829217	Wanasinghe et al. (2018)
*Neophaeosphaeria agaves*	CPC 21264	KF777227	N/A	N/A	N/A	Crous et al. (2013)
*Neophaeosphaeria filamentosa*	CBS 102202	GQ387577	GU371773	GQ387516	GU349084	de Gruyter et al. (2013)
*Neophaeosphaeria phragmiticola*	KUMCC 16-0216	MG837009	N/A	NG_065735	MG838020	Hyde et al. (2018)
*Neopyrenochaeta acicola*	CBS 812.95	GQ387602	LT623271	NG_065567	N/A	de Gruyter et al. (2010); Valenzuela-Lopez et al. (2018)
*Neopyrenochaeta cercidis*	MFLU 18-2089	MK347932	MK434908	MK347823	N/A	Jayasiri et al. (2019)
*Neoroussoella bambusae*	MFLUCC 11-0124	KJ474839	KJ474856	N/A	KJ474848	Liu et al. (2014)
*Nigrograna fuscidula*	CBS 141556	KX650550	N/A	N/A	KX650525	Jaklitsch and Voglmayr (2016)
*Nigrograna mackinnonii*	CBS 674.75	GQ387613	KF015703	NG_061081	KF407986	de Gruyter et al. (2010); Ahmed et al. (2014)
*Nigrograna obliqua*	CBS 141475	KX650558	KX650579	KX650512	KX650530	Jaklitsch and Voglmayr (2016)
*Occultibambusa bambusae*	MFLUCC 13-0855	KU863112	KU940170	KU872116	KU940193	Dai et al. (2016)
*Occultibambusa jonesii*	GZCC 16-0117	KY628322	KY814758	KY628324	KY814756	Zhang et al. (2017)
*Ohleria modesta*	OM	KX650563	KX650583	KX650513	KX650534	Jaklitsch et al. (2016)
*Ohleria modesta*	MGC	KX650562	KX650582	N/A	KX650533	Jaklitsch et al. (2016)
*Palawania thailandensis*	MFLU 16-1871	KY086494	N/A	N/A	N/A	Mapook et al. (2016a)
*Palawania thailandensis*	MFLUCC 14-1121	NG_241882	KY086496	NG_242378	N/A	Mapook et al. (2016a)
*Parabambusicola bambusina*	KH 139	AB807537	N/A	AB797247	AB808512	Tanaka et al. (2015)
*Paradictyoarthrinium diffractum*	MFLUCC 13-0466	KP744498	KX437764	KP753960	N/A	Liu et al. (2015)
*Paradictyoarthrinium hydei*	MFLUCC 17-2512	NG_067558	MG780232	NG_065757	N/A	Liu et al. (2018b)
*Paralophiostoma hysterioides*	PUFNI 17617	MT912850	MT926117	MT914175	N/A	Hongsanan et al. (2020a)
*Paramycoleptodiscus albizziae*	CBS 141320	KX228330	MK492737	N/A	MK495979	Hernández-Restrepo et al. (2019)
*Parapyrenochaeta protearum*	CBS 131315	JQ044453	LT717683	N/A	N/A	Valenzuela-Lopez et al. (2018)
*Paratrimmatostroma kunmingensis*	KUN-HKAS 102224A	MK098196	N/A	MK098204	MK098208	Jayawardena et al. (2022)
*Periconia delonicis*	MFLUCC 17-2584	NG_068611	MK434901	NG_065770	MK360071	Jayasiri et al. (2019)
*Periconia pseudodigitata*	KT 1395	AB807564	N/A	AB797274	AB808540	Tanaka et al. (2015)
*Phaeoseptum terricola*	MFLUCC 10-0102	MH105779	MH105782	NG_065749	MH105781	Hyde et al. (2018)
*Phaeosphaeria oryzae*	CBS 110110	KF251689	KF252193	GQ387530	ON419509	de Gruyter et al. (2010); Quaedvlieg et al. (2013); Ashrafi et al. (2023)
*Phaeosphaeriopsis triseptata*	MFLUCC 13-0271	KJ522479	KJ522485	KJ522484	MG520919	Thambugala et al. (2014)
*Phaeotrichum benjaminii*	CBS 541.72	AY004340	DQ677946	AY016348	DQ677892	Lumbsch et al. (2005); Schoch et al. (2006a)
*Pleomonodictys capensis*	CBS 968.97	KY853521	N/A	N/A	N/A	Hernández-Restrepo et al. (2017)
*Pleomonodictys descalsii*	FMR 12716	KY853522	N/A	N/A	N/A	Hernández-Restrepo et al. (2017)
*Preussia funiculata*	CBS 659.74	GU301864	GU371799	GU296187	GU349032	de Gruyter et al. (2009)
*Prosthemium betulinum*	CBS 279.74	MH872591	KT216532	DQ678027	DQ677923	Schoch et al. (2006a); Vu et al. (2019)
*Prosthemium stellare*	CBS 126964	MH875800	N/A	AB553650	N/A	Tanaka et al. (2010); Vu et al. (2019)
*Protofenestella ulmi*	FP5 = CBS 143000	MF795791	MF795833	N/A	MF795879	Jaklitsch et al. (2018)
*Psedotubeufia laxispora*	GZCC 22-2011	OR030831	OR046682	N/A	OR046675	Ma et al. (2023a)
*Pseudoastrosphaeriella bambusae*	MFLUCC 11-0205	KT955475	KT955414	KT955455	KT955437	Phookamsak et al. (2015)
*Pseudoastrosphaeriella longicolla*	MFLUCC 11-0171	KT955476	KT955420	N/A	KT955438	Phookamsak et al. (2015)
*Pseudoastrosphaeriella thailandensis*	MFLUCC 11-0144	KT955478	KT955416	KT955457	KT955440	Phookamsak et al. (2015)
*Pseudoberkleasmium chiangmaiense*	MFLUCC 17-1809	MK131260	N/A	N/A	MK131261	Hyde et al. (2019)
*Pseudoberkleasmium pandanicola*	KUMCC 17-0178	MH260304	N/A	MH260344	N/A	Tibpromma et al. (2018a)
*Pseudocoleodictyospora tectonae*	MFLUCC 12-0385	KU764709	KU712491	NG_061232	N/A	Doilom et al. (2017)
*Pseudocoleodictyospora thailandica*	MFLUCC 12-0565	KU764701	KU712494	NG_062417	N/A	Doilom et al. (2017)
*Pseudolophiotrema elymicola*	KT 1450	LC194381	LC194473	LC194339	LC194418	Hashimoto et al. (2017a)
*Pseudomassarina clematidis*	MFLU 16-0493	NG_073850	MT394700	NG_070663	MT394644	Thambugala et al. (2015)
*Pseudopalawania siamensis*	MFLUCC 17-1476a	N/A	N/A	MT137789	MT136752	Mapook et al. (2020b)
*Pseudopalawania siamensis*	MFLUCC 17-1476b	NA	N/A	MT137790	N/A	Mapook et al. (2020b)
*Pseudopalawaniella woodwardiae*	KUNCC 23-13877	PV862394	PV948866	PV862381	PV948879	This study
*Pseudopyrenochaeta lycopersici*	CBS 306.65	EU754205	LT717680	NG_062728	N/A	Valenzuela-Lopez et al. (2018)
*Pyrenochaetopsis leptospora*	CBS 101635	GQ387627	LT623282	NG_063097	MF795881	de Gruyter et al. (2010)
*Pyrenochaetopsis tabarestanensis*	IBRC M 30051	KF803343	N/A	NG_065034	N/A	Papizadeh et al. (2017)
*Pyrenophora phaeocomes*	AFTOL-ID 283	NG_027575	DQ497614	JN940960	DQ497607	Goonasekara et al. (2020）
*Quadrisporella heveae*	MFLUCC 18-0308	OL782057	OL828755	N/A	OL875101	Senwanna et al. (2021)
*Quercicola fusiformis*	MFLUCC 18-0479	MK348009	MK434864	MK347898	MK360085	Jayasiri et al. (2019)
*Quercicola guttulospora*	MFLUCC 18-0481	MK348010	N/A	MK347899	MK360086	Jayasiri et al. (2019)
*Quixadomyces cearensis*	HUEFS 238438	MG970695	N/A	N/A	N/A	Crous et al. (2018c)
*Ramusculicola thailandica*	MFLUCC 13-0284	KP888647	N/A	KP899131	KR075167	Thambugala et al. (2015)
*Roccella fuciformis*	Tehler 8171	FJ638979	N/A	N/A	N/A	Zhang et al. (2019)
*Rostriconidium pandanicola*	KUMCC 17-0176	NG_068830	MH412759	MH260358	MH412781	Tibpromma et al. (2018a)
*Roussoella nitidula*	MFLUCC 11-0634	KJ474842	KJ474858	N/A	KJ474851	Liu et al. (2014)
*Salsuginea phoenicis*	MFLU 19-0015	MK405280	N/A	N/A	MK404650	Jones er al. (2019)
*Salsuginea ramicola*	KT 2597.2	GU479801	GU479834	GU479768	GU479862	Suetrong et al. (2009)
*Setoapiospora thailandica*	AND3	OL457707	N/A	OL700220	OL998895	de Silva et al. (2022)
*Setoapiospora thailandica*	MFLUCC 17-1426	NG_068914	N/A	NG_068420	MN648731	Hyde et al. (2020)
*Shiraia bambusicola*	GZAAS2.629	KC460980	N/A	N/A	N/A	Liu et al. (2013)
*Sirodesmium olivaceum*	CBS 395.59	GU250894	GU250947	GU250915	N/A	Ruibal et al. (2009)
*Sporormia fimetaria*	Gr.81.194	GQ203729	N/A	N/A	N/A	Kruys and Wedin (2009)
*Stemphylium vesicarium*	CBS 191.86	GU238160	DQ247794	GU238232	DQ471090	Schoch et al. (2006b); Spatafora et al. (2006); Aveskamp et al. (2010)
*Striatiguttula nypae*	MFLUCC 18-0265	MK035992	MK034440	MK035977	MK034432	Zhang et al. (2019)
*Striatiguttula phoenicis*	MFLUCC 18-0266	MK035995	MK034442	MK035980	MK034435	Zhang et al. (2019)
*Subglobosporium tectonae*	MFLUCC 12-0393	KU764703	KU712485	NG_061233	N/A	Doilom et al. (2017)
*Subplenodomus violicola*	CBS 306.68	MH870849	N/A	GU238231	N/A	Aveskamp et al. (2010); Vu et al. (2019)
*Sulcatispora acerina*	KUMCC 21-0821	ON009112	ON009294	ON009096	ON009271	Wanasinghe et al. (2022)
*Sulcatispora acerina*	KT 2982	LC014610	N/A	LC014605	LC014615	Tanaka et al. (2015)
*Sulcosporium thailandica*	MFLUCC 12-0004	KT426563	N/A	KT426564	N/A	Ariyawansa et al. (2015a)
*Synnematospora pronephrii*	KUNCC 23-13965	PV862395	N/A	PV862382	PV948880	This study
*Teichospora trabicola*	C134	KU601591	KU601600	N/A	KU601601	Jaklitsch et al. (2016)
*Tetraploa bambusae*	KUMCC 21-0844	ON077067	N/A	ON077073	ON075061	Phookamsak et al. (2022)
*Tetraploa cylindrica*	KUMCC 20-0205	MT893204	N/A	MT893203	N/A	Liao et al. (2022)
*Tetraploa dashaoensis*	KUMCC 21-0010	OL473555	N/A	OL473556	N/A	Jayawardena et al. (2022)
*Thyridaria broussonetiae*	TB1 = CBS 141481	KX650568	KX650586	KX650515	KX650539	Jaklitsch and Voglmayr (2016)
*Torula aquatica*	MFLUCC 16-1115	MG208146	MG207977	N/A	N/A	Su et al. (2018)
*Torula pluriseptata*	MFLUCC 14-0437	KY197855	KY197869	KY197862	KY197875	Li et al. (2017)
*Trematosphaeria grisea*	CBS 332.50	NG_057979	KF015720	NG_062930	KF015698	Ahmed et al. (2014)
*Trematosphaeria pertusa*	CBS 122368	NG_057809	FJ795476	FJ201991	KF015701	Ahmed et al. (2014)
*Trichodelitschia munkii*	Kruys 201 (UPS)	DQ384096	N/A	DQ384070	N/A	Kruys et al. (2006)
*Tubeufia muriformis*	GZCC 22-2039	OR030836	OR046686	N/A	OR046680	Ma et al. (2023b)
*Tzeanania taiwanensis*	NTUCC 17-006	MH461121	MH461129	MH461127	MH461131	Ariyawansa et al. (2018)
*Verruculina enalia*	BCC 18402	GU479803	GU479836	GU479771	GU479864	Suetrong et al. (2009)
*Westerdykella angulata*	CBS 610.74	NG_057754	N/A	NG_062146	GU371821	Kruys et al. (2006)
*Wicklowia aquatica*	CBS 125634	MH875044	N/A	NG_061099	N/A	Schoch et al. (2009); Vu et al. (2019)
*Wicklowia submersa*	MFLUCC 18-0373	MK637644	N/A	MK637643	N/A	Boonmee et al. (2019)
*Xenoberkleasmium chiangraiense*	GZAAS 24-0051	PP657340	PP887797	N/A	N/A	Liu et al. (2024a)
*Xenoberkleasmium pandani*	KUNCC 23-13876	PV862396	PV948867	N/A	PV948881	This study
*Xenoberkleasmium pandani*	KUNCC 23-13878	PV862397	PV948868	PV862383	PV948882	This study
*Xenoberkleasmium pandani*	KUNCC 23-14012	PV862398	PV948869	PV862384	PV948883	This study
*Xenopleopunctum guizhouense*	KUNCC 23-13881	PV862400	PV948871	PV862386	PV948885	This study
*Xenopleopunctum guizhouense*	KUNCC 23-13880	PV862401	PV948872	PV862387	PV948886	This study
*Xenopleopunctum guizhouense*	KUNCC 23-13882	PV862402	PV948873	PV862388	PV948887	This study
*Xenopleopunctum sporodochiale*	GZCC 23-0742	PV862403	PV948874	PV862389	PV948888	This study
*Xenopyrenochaetopsis pratorum*	CBS 445.81	GU238136	KT389671	NG_062792	N/A	Aveskamp et al. (2010)

ML and BI analyses were performed through the CIPRES Science Gateway (Miller et al. 2010). ML analysis was conducted with RAxML-HPC v. 8.2.12 (Stamatakis 2014) using a GTRGAMMA approximation with rapid bootstrap analysis followed by 1000 bootstrap replicates. BI analysis was performed in a likelihood framework implemented on XSEDE 3.2.7a (Ronquist et al. 2012). Bayesian posterior probabilities (PPs) (Rannala and Yang 1996; Huelsenbeck 2001) were evaluated based on Markov Chain Monte Carlo (MCMC) sampling. Four simultaneous Markov chains were run for 100,000,000 generations, or the searches were stopped when the average standard deviation of split frequencies was below 0.01 (stopval = 0.01). Trees were sampled every 1000 generations, yielding 100,000 trees in total. The first 25% of trees were set as burn-in and discarded. The remaining trees were used to calculate PPs (Larget and Simon 1999).

The phylogenetic tree was visualized using FigTree v. 1.4.4 (Rambaut et al. 2014), and the layouts were reorganized online following the methods described in Xie et al. (2023) and finalized with Adobe Illustrator CS6 (Adobe Systems, USA).

## ﻿Results

### ﻿Phylogenetic result

**Figure 1.**
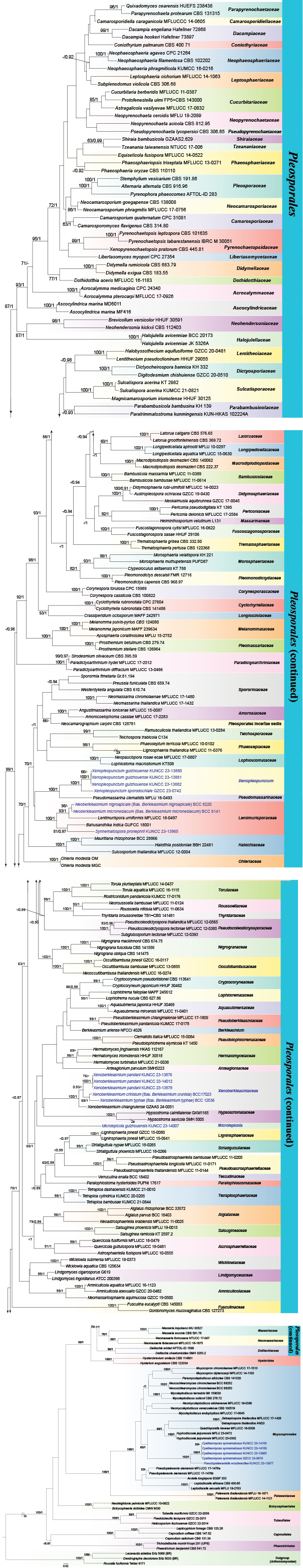
Phylogram generated from maximum likelihood analysis based on combined LSU, *RPB2*, SSU, and *tef1-α* sequence data in *Dothideomycetes*. Bootstrap support values for maximum likelihood (ML) greater than 60% and Bayesian posterior probabilities (PPs) greater than 0.90 are indicated near the nodes as ML/PP. The tree is rooted with three species of *Arthoniomycetes*, including *Dendrographa decolorans* (Ertz 5003), *Lecanactis abietina* (Ertz 5068), and *Roccella fuciformis* (Tehler 8171). The new taxa and new combinations are shown in blue.

A combined dataset of LSU, *RPB2*, SSU, and *tef1-α* sequence data was used to evaluate the phylogenetic placement of the new taxa in *Pleosporales* and *Muyocopronales*, *Dothideomycetes*. The concatenated sequence matrix comprised 234 taxa with representative taxa of *Pleosporales* and *Muyocopronales* and several major groups in *Dothideomycetes*, with three species of *Arthoniomycetes* as outgroup taxa. After alignment, the dataset contained 3,785 characters (LSU: 1–853; *RPB2*: 854–1,870 bp; SSU: 1,871–2,879, *tef1-α*: 2,880–3,785), including 2,589 distinct alignment patterns, with 32.15% comprising undetermined characters or gaps, 1,717 parsimony-informative sites, 426 singleton sites, and 1,642 constant sites. The total tree length is 27.938381. The best-scoring RAxML tree is shown in Fig. 1, with a final likelihood value of −112695.550615.

Our 14 fresh collections representing seven species are placed in two orders (*Muyocopronales* and *Pleosporales*). Among these, five isolates represent two distinct, independent lineages identified as *Cyatheomyces synnematosus* and *Pseudopalawaniella woodwardiae* in *Muyocopronaceae*, *Muyocopronales*. Notably, these two lineages share a sister relationship with high support (100% ML/1.00 PP, Fig. 1).

The analysis confirms that *Lentimurisporaceae* is a monophyletic family within *Pleosporales*. *Synnematospora pronephrii*, representing a newly proposed genus, is sister to the genus *Bahusandhika* with high support. Another new genus, *Neoberkleasmium*, introduced in *Lentimurisporaceae*, forms a distinct clade that includes two new combinations transferred from *Berkleasmium*.

The phylogenetic tree reveals that our three new collections representing *Xenoberkleasmium pandani* (≡ *Berkleasmium pandani*) and two additional *Berkleasmium* species cluster with *X. chiangraiense*, the type species of *Xenoberkleasmium*. These four species form an independent, monophyletic clade with strong support (100% ML/1.00 PP, Fig. 1), representing a new family, *Xenoberkleasmiaceae*, which is closely related to *Hypsostromataceae*.

A new fresh collection is identified as a new monotypic genus, *Microlepicola*, within *Pleosporales* genera incertae sedis. This clade is basal to several families, including *Anteagloniaceae*, *Aquasubmersaceae*, *Cryptocoryneaceae*, *Hypsostromataceae*, *Lophiotremataceae*, *Hermatomycetaceae*, *Pseudoberkleasmiaceae*, *Pseudolophiotremataceae*, and *Xenoberkleasmiaceae*.

The last four strains form a well-supported monophyletic clade, representing a new lineage within *Pleosporales*. The phylogenetic analysis also indicates that this clade is closely related to *Pseudomassarina* and can be recognized as a novel genus, *Xenopleopunctum*, comprising two new species, *X. guizhouense* and *X. sporodochiale*.

### ﻿Taxonomy

#### ﻿ Muyocopronales

Taxon classificationFungiDothideomycetesMuyocopronales

Mapook, Boonmee & K.D. Hyde, Phytotaxa 265(3): 230 (2016)

2424050E-1772-529C-B83D-A79EBCEFDBE7

Muyocopronaceae K.D. Hyde, Fungal Diversity 63: 164 (2013).

##### Notes.

*Muyocopronaceae* was initially established by Luttrell (1951; Nom. inval., Art. 39.1, Melbourne) and was validly introduced by Hyde et al. (2013) to accommodate *Muyocopron*. This family currently comprises 12 genera, viz., *Arxiella*, *Hyphodiscosia*, *Leptodiscella*, *Muyocopron*, *Muyocopromyces*, *Mycoleptodiscus*, *Neocochlearomyces*, *Neomycoleptodiscus*, *Paramycoleptodiscus*, *Pseudopalawania*, *Quadrisporella*, and *Setoapiospora* (Hyde et al. 2020; Mapook et al. 2020a; Xu et al. 2024).

#### ﻿ Cyatheomyces

Taxon classificationFungiMuyocopronalesMuyocopronaceae

J.Y. Zhang, Y.Z. Lu & K.D. Hyde gen. nov.

2B96A388-9265-5EF1-9607-22237699B130

Index Fungorum: IF904131

##### Etymology.

The genus name refers to the host genus *Cyathea*, combined with “myces” for fungi.

##### Type species.

*Cyatheomyces synnematosus* J.Y. Zhang, Y.Z. Lu & K.D. Hyde.

##### Description.

Sexual morph: Undetermined. Asexual morph: Hyphomycetous. ***Colonies*** on natural substrate effuse, scattered or aggregated, brown, with masses of conidia at the apex. ***Mycelium*** partly immersed, partly superficial, composed of branched, septate, hyaline to pale brown hyphae. ***Conidiophores*** macronematous, synnematous, erect, cylindrical, thick-walled, aseptate, brown to hyaline towards the apex. ***Conidiogenous cells*** monoblastic to polyblastic, integrated, terminal, hyaline or pale brown, sympodial, often with denticles. ***Conidia*** oblong to obovoid to clavate, septate, hyaline, rarely pale brown.

#### ﻿ Cyatheomyces synnematosus

Taxon classificationFungiMuyocopronalesMuyocopronaceae

J.Y. Zhang, Y.Z. Lu & K.D. Hyde sp. nov.

568E1450-BB66-5B85-9D3E-9C78F7AEF7FC

Index Fungorum: IF904132

Figs 2, 3

##### Etymology.

The species epithet refers to the synnematous conidiomata.

##### Diagnosis.

Similar to *Phaeoisaria* and *Rhamphoriopsis*, but *C. synnematosus* differs from them by its various conidia.

##### Holotype.

HKAS 129699.

##### Description.

**Figure 2.**
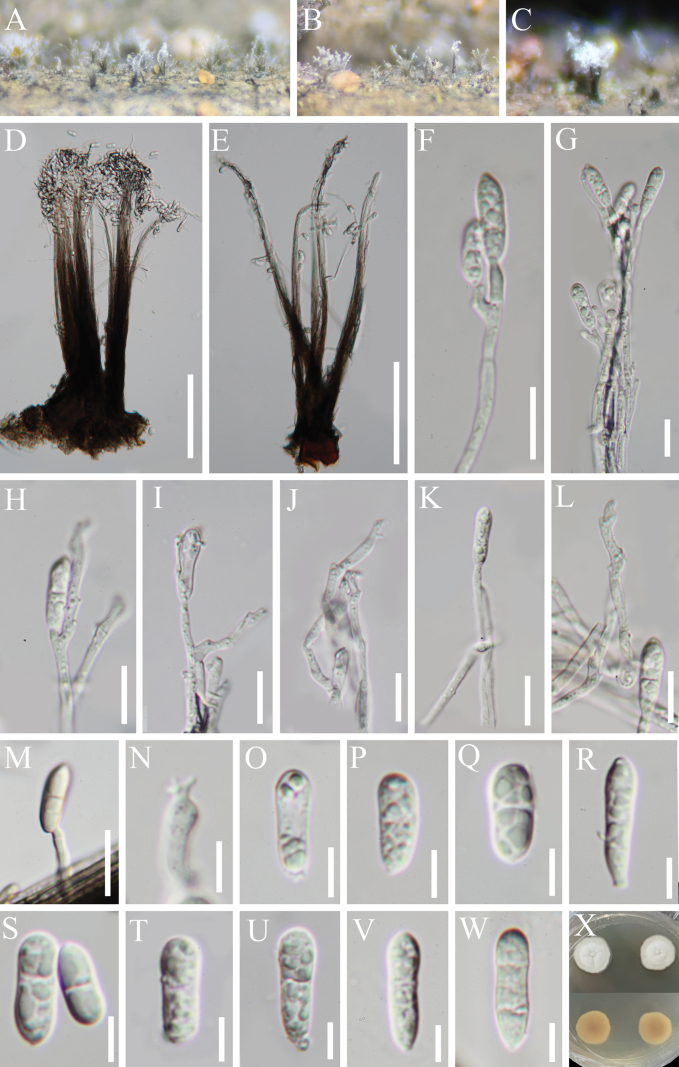
*Cyatheomyces synnematosus* (HKAS 129699, holotype). **A–C** Colonies on host substrate; **D, E** Conidiophores; **F–M** Conidiogenous cells; **N** Conidiogenous loci; **O–W** Conidia; **X** Pure culture from above and below. Scale bars: 100 µm (**D, E**); 10 µm (**F–M**); 5 µm (**N–W**).

**Figure 3.**
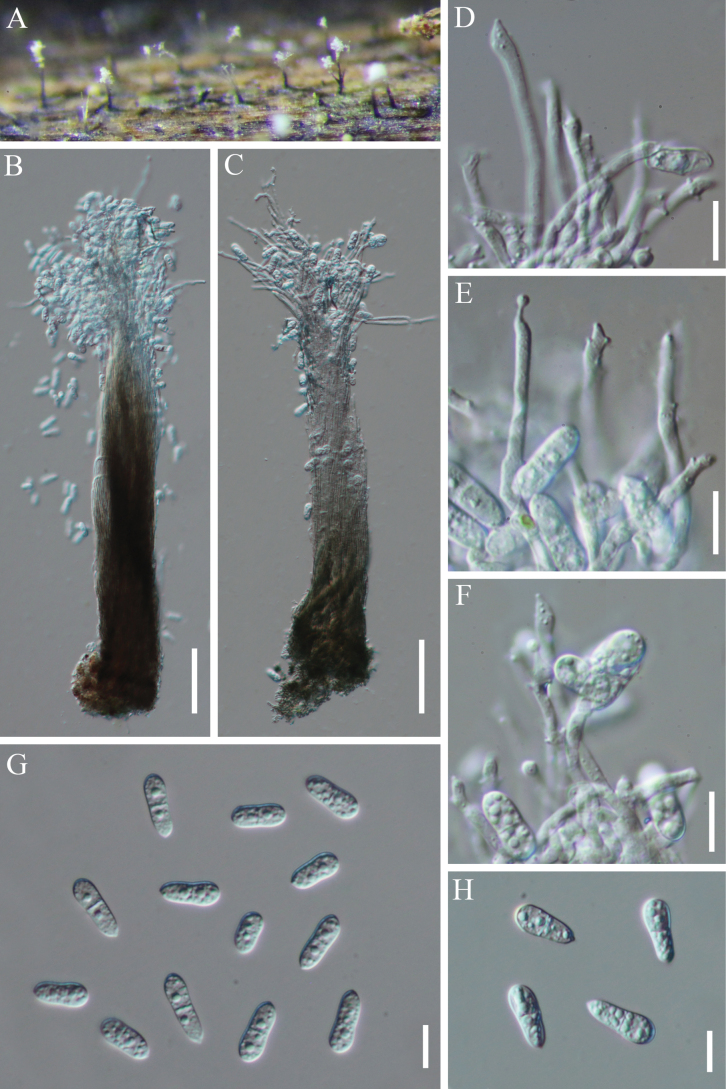
*Cyatheomyces synnematosus* (HKAS 129695, paratype). **A** Colonies on host substrate; **B, C** Conidiophores with conidia; **D–F** Conidiogenous cells with conidia; **G, H** Conidia. Scale bars: 50 µm (**B, C**); 10 µm (**D–H**).

Sexual morph: Undetermined. Asexual morph: Hyphomycetous. ***Colonies*** on natural substrate effuse, scattered or aggregated, brown, with white conidial masses at the apex. ***Mycelium*** partly immersed, partly superficial, composed of branched, thick-walled, septate, hyaline to pale brown hyphae. ***Synnemata*** up to 78 μm wide near the base, dark brown to hyaline towards the apex, with upper parts of conidiophores slightly splaying out as a flared head. ***Conidiophores*** macronematous, synnematous, erect, cylindrical, often flexuous at the conidiogenous region, thick-walled, aseptate, brown to hyaline towards the apex, 158–362 μm × 1.3–2.5 μm (*x̄* = 281 × 1.8, n = 20). Two modes of development during conidiogenesis; ***Conidiogenous cells****type 1* (Figs 2F–M, 3F) monoblastic, integrated, terminal, hyaline or subhyaline, 1.3–2.5 μm wide; ***Conidiogenous cells****type 2* (Figs 2N, 3D–F) polyblastic, integrated, terminal, sympodia, with inconspicuous denticles, hyaline or subhyaline, 1.5–2.8 μm wide near the conidiogenous loci. ***Conidia*** various, oblong to obovoid to clavate, aseptate when young, 1(–2)-septate when mature, sometime indistinct, not constricted at the septum, guttulate, hyaline, rarely pale brown, 11–15 × (2.5–)3.7–5 µm (*x̄* = 13 × 4.4 µm, n = 25).

##### Culture characteristics.

Conidia germinating on WA within 15 h and germ tube produced from conidia. Colonies growing on PDA, grown slowly, reaching 11–13 mm diameter after one month at room temperature (ca. 26 °C), circular with entire margin, flat with a protuberance in the center, veined, from which several indentations extend outwards, and cut into fan shapes at the surface, white form above; brown form below, and not producing pigmentation in cultures.

##### Material examined.

CHINA • Guizhou Province, Zunyi City, Chishui County, Hushi Town, Chishui Alsophila Natural Reserve, on dead frond stalks of *Cyathea* sp. (*Cyatheaceae*), 27 July 2022, J.Y. Zhang, CX4 (HKAS 129699, holotype; GZAAS 23-0669, isotype), ex-type living culture, KUNCC 23-13865; • ibid., 22 September 2019, J.Y. Zhang, C4 (HKAS 129695 = GZAAS 23-0776, paratype), living culture, GZCC 23-0670; • ibid., 14 April 2023, J.Y. Zhang, ZY16 (HKAS 129859, paratype), living culture, KUNCC 23-14156; • ibid., ZY18 (HKAS 147019, paratype), living culture, KUNCC 23-14159.

##### Additional sequence.

KUNCC 23-13865: ITS (PV862363); GZCC 23-0670: ITS (PV862364); KUNCC 23-14156: ITS (PV862365); KUNCC 23-14159: ITS (PV862366).

##### Notes.

Four new strains formed a phylogenetically distinct lineage within *Muyocopronaceae* and are described as a new genus, *Cyatheomyces*. *Cyatheomyces synnematosus* has a unique morphology, characterized by macronematous, synnematous conidiophores, which notably distinguishes it from other genera in this family, which typically have solitary, micro- or macronematous, mononematous conidiophores (Crous et al. 2018a; Hernández-Restrepo et al. 2019; Xu et al. 2024). The morphological characteristics are similar to species in *Phaeoisaria* and *Rhamphoriopsis* (Hyde et al. 2018, 2019; Yang et al. 2023). However, phylogenetic analysis showed that *Phaeoisaria* was placed in *Pleurotheciaceae*, *Pleurotheciales*, *Sordariomycetes*, and *Rhamphoriopsis* in *Rhamphoriaceae*, *Rhamphoriales*, *Sordariomycetes* (Hyde et al. 2024). Comparatively, *Cyatheomyces* is assigned to *Muyocopronaceae* (*Muyocopronales*, *Dothideomycetes*) based on evidence from morphology and phylogeny.

#### ﻿ Pseudopalawaniella

Taxon classificationFungiMuyocopronalesMuyocopronaceae

J.Y. Zhang, K.D. Hyde & Y.Z. Lu gen. nov.

7CEB74DD-5B42-5B8B-B0CE-1BE32C3C8CA3

Index Fungorum: IF904133

##### Etymology.

The genus name refers to the similarity to *Pseudopalawania*.

##### Type species.

*Pseudopalawaniella woodwardiae* J.Y. Zhang, K.D. Hyde & Y.Z. Lu.

##### Description.

Sexual morph: ***Ascomata*** superficial, solitary or scattered, sub-carbonaceous to carbonaceous, flattened or raised, with a poorly developed basal layer and an irregular margin, dark brown to black. ***Peridium*** composed of dark brown or black cells of *textura angularis*. ***Hamathecium*** cylindrical to filiform, branched, septate, pseudoparaphyses, hyaline. ***Asci*** 8-spored, bitunicate, fissitunicate, cylindric-clavate, apically rounded, straight or slightly curved, sessile or short pedicellate. ***Ascospores*** overlapping, irregularly arranged, ellipsoid to broadly fusiform, with rounded ends, straight or slightly curved, 1-septate, constricted at the septum, guttulate, hyaline. Asexual morph: Undetermined.

#### ﻿ Pseudopalawaniella woodwardiae

Taxon classificationFungiMuyocopronalesMuyocopronaceae

J.Y. Zhang, Y.Z. Lu & K.D. Hyde sp. nov.

9AFA7F93-6024-55D0-9DA9-CF58584976AF

Index Fungorum: IF904134

Fig. 4

##### Etymology.

The species epithet refers to this fungal host, “*Woodwardia japonica*”.

**Figure 4.**
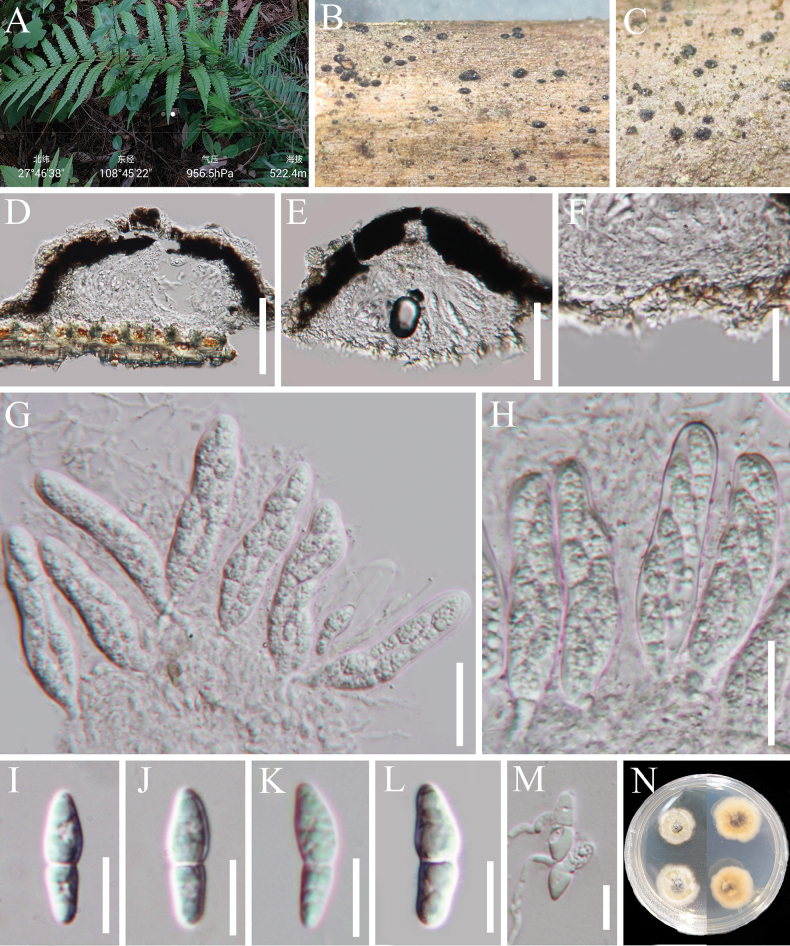
*Pseudopalawaniella woodwardiae* (HKAS 129705, holotype). **A** The host; **B, C** Colonies; **D, E** Sections through ascomata; **F** Peridium mixed with host substrate at the base of an ascoma; **G, H** Asci and pseudoparaphyses; **I–L** Ascospores; **M** Germinated ascospores; **N** Pure culture from above and below. Scale bars: 50 μm (**D, E**); 20 μm (**F–H**); 10 μm (**I–M**).

##### Diagnosis.

Differs from *Pseudopalawania* species in its shapes of ascomata (Raised *vs.* Flattened), asci (asci without distinct ocular chambers *vs.* asci with distinct ocular chambers) and ascospores (ellipsoid to broadly fusiform ascospores with obtuse ends *vs.* fusiform to inequilateral ascospores with pointed ends).

##### Holotype.

HKAS 129705

##### Description.

Sexual morph: ***Ascomata*** superficial, solitary or scattered, raised, rarely flattened, sub-carbonaceous to carbonaceous, appearing as circular, flattened, covering the host, with a poorly developed basal layer and an irregular margin, dark brown to black spots, 139–189 µm × (73–)85–111.5 µm high (*x̄* = 163 × 97 µm, n = 15). ***Ostioles*** central. ***Peridium*** comprising dark brown or black cells of *textura angularis*, 13–22.5 µm wide. ***Hamathecium*** cylindrical to filiform, septate, pseudoparaphyses, hyaline, 1–2(–2.6) µm wide. ***Asci*** 8-spored, bitunicate, fissitunicate, cylindric-clavate, straight or slightly curved, sessile or inconspicuous pedicellate, apically rounded, with an inconspicuous ocular chamber, hyaline, 46–53 × 9.5–12 µm (*x̄* = 47.5 × 10.5 µm, n = 20). ***Ascospores*** overlapping, 2–3-seriate, ellipsoid to broadly fusiform, inequilateral, with obtuse ends, straight or slightly curved, 1-septate, 3.5–6.5 wide at septum, constricted at the septum, with a slightly small lower cell, hyaline or subhyaline, 14.6–19.7 × 4.7–6.5 µm (*x̄* = 17 × 5 µm, n = 20). Asexual morph: Undetermined.

##### Culture characteristics.

Ascospores germinating on WA within 15 h at 26 °C. Colonies on PDA, circular with slight wavy margin, flat with protuberance and wrinkle in the center, veined, yellowish white with ashen in the center from above; beige with dark brown in the middle from below, and not producing pigmentation in cultures.

##### Material examined.

CHINA • Guizhou Province, Tongren City, Jiangkou County, (27°46'38"N, 108°45'22"E), on dead leaf axis of *Woodwardia japonica* (*Blechnaceae*) in a forest near the roadside, 21 May 2022, J.Y. Zhang, F31-3 (HKAS 129705, holotype; GZAAS 23-0673, isotype), ex-type living culture KUNCC 23-13877.

##### Additional sequence.

ITS (PV862367).

##### Notes.

*Pseudopalawaniella* resembles *Pseudopalawania* in superficial, sub-carbonaceous to carbonaceous ascomata covering the host, cylindric-clavate asci with an ocular chamber, and hyaline, 1-septate ascospores (Mapook et al. 2020b). However, they are phylogenetically distinct and also differ in the shapes of their ascomata (raised vs. flattened), asci (asci without distinct ocular chambers vs. asci with distinct ocular chambers), and ascospores (ellipsoid to broadly fusiform ascospores with obtuse ends vs. fusiform to inequilateral ascospores with pointed ends). Multigene phylogenetic analysis shows that *Pseudopalawaniella woodwardiae* forms an independent clade within *Muyocopronaceae*, sister to *Cyatheomyces synnematosus*, with good bootstrap support (100% ML/1.00 PP, Fig. 1). However, we were unable to compare the morphological characteristics of these two species, as *Pseudopalawaniella woodwardiae* presents a sexual morph in nature, whereas *Cyatheomyces synnematosus* exhibits an asexual morph. Attempts were made to culture the asexual morph in *Pseudopalawaniella woodwardiae* and the sexual morph in *Cyatheomyces synnematosus*, but these attempts failed. Therefore, there is no morphological evidence to prove that the two species belong to the same genus. Furthermore, a comparison of nucleotide base pairs of LSU, ITS, *RPB2*, and *tef1-α* between *Pseudopalawaniella woodwardiae* (HKAS 129705) and *Cyatheomyces synnematosus* (HKAS 129699) showed 27/833 bp (3.2%, including 3 gaps), 97/839 bp (11.6%, including 42 gaps), 95/1079 bp (8.8%, without gaps), and 45/991 bp (4.5%, without gaps) differences. Hence, to avoid taxonomic confusion, we introduce *Pseudopalawaniella* as a new genus in *Muyocopronaceae (Muyocopronales)*. Further morphological investigations, together with more collections and molecular data, are needed to clarify the status of these two genera.

#### ﻿ Pleosporales

Taxon classificationFungiDothideomycetesPleosporales

Luttr. ex M.E. Barr, Prodr. Cl. Loculoasc. (Amherst): 67 (1987).

821416EF-E08F-5529-8648-911ECFCC5B12

Lentimurisporaceae N.G. Liu, Jian K. Liu & K.D. Hyde, Cryptog. Mycol. 39(2): 270 (2018).

##### Notes.

*Lentimurisporaceae* was introduced as a new pleosporalean family by Liu et al. (2018) to accommodate *Bahusandhika*, *Lentimurispora*, and two *Berkleasmium* species based on morphology, phylogeny, and divergence time estimates. Members of *Lentimurisporaceae* are dematiaceous hyphomycetes, which are characterized by punctiform colonies, sporodochial conidiophores, blastic conidiogenous cells, muriform or fusiform, cylindrical or rhomboidal conidia (Liu et al. 2018a; Hongsanan et al. 2020a). In this study, we introduce two new genera (*Neoberkleasmium* and *Synnematospora*) and emend the concept of *Lentimurisporaceae* to include synnematous conidiophores.

#### ﻿ Neoberkleasmium

Taxon classificationFungiPleosporalesLentimurisporaceae

J.Y. Zhang, Y.Z. Lu & K.D. Hyde gen. nov.

A94EF615-FD32-5DC8-806F-24FD2FDBD7C9

Index Fungorum: IF904137

##### Etymology.

The genus name refers to the similar genus *Berkleasmium*.

##### Type species.

*Neoberkleasmium nigroapicale* (Bussaban, Lumyong, P. Lumyong, McKenzie & K.D. Hyde) J.Y. Zhang, K.D. Hyde & Y.Z. Lu.

##### Description.

Sexual morph: Undetermined. Asexual morph: hyphomycetous. ***Colonies*** on natural substrate superficial, effuse, scattered or aggregated, sporodochial, pulvinata, punctiform, black. ***Mycelium*** partly immersed, partly superficial, composed of branched, septate, subhyaline to brown hyphae. ***Conidiophores*** macronematous, septate, hyaline to pale brown. ***Conidiogenous cells*** blastic, integrated, terminal, hyaline. ***Conidia*** acrogenous, solitary, cylindrical to broadly clavate, thick-walled, muriform, constricted at septa, brown.

##### Notes.

*Neoberkleasmium* is introduced as a segregated genus from *Berkleasmium* to accommodate *Berkleasmium micronesiacum* and *B. nigroapicale*. *Berkleasmium* species have been associated with helicosporous fungi and were placed in *Tubeufiaceae*, *Tubeufiales* (Lu et al. 2017; Tibpromma et al. 2017). In comparison, *Neoberkleasmium* is a member of *Lentimurisporaceae*, *Pleosporales*. Morphologically, *Neoberkleasmium* fits well with the concept of *Lentimurisporaceae* and resembles *Lentimurispora* in having sporodochial, brown to black conidiomata, monoblastic conidiogenous cells, and muriform, dematiaceous conidia (Table 2). However, *Neoberkleasmium* is distinguished from *Lentimurispora* by its cylindrical to broadly clavate conidia, often darkened at the upper part, whereas *Lentimurispora* produces conidia that are lenticular with dark brown central cells and pale-colored peripheral cells.

**Table 2.** Morphological comparison of four accepted genera in *Lentimurisporaceae*.

Genera	Conidiophores	Conidiogenous cells	Conidia	Reference(s)
* Bahusandhika *	Sporodochial, micronematous	Spherical, ovoid, or ampulliform	Fusiform, cylindrical, or rhomboidal; 1–3-septate; catenate; brown	Pratibha et al. (2014); Crous et al. (2015); Crane and Miller (2016)
* Lentimurispora *	Sporodochial, micronematous	Inverted vase-like, clavate	Lenticular; muriform, dark brown central cells and subhyaline to pale brown peripheral cells	Liu et al. (2018a)
* Neoberkleasmium *	Sporodochial, macronematous	Cylindrical	Cylindrical to broadly clavate; muriform; brown	Matsushima (1981); Bussaban et al. (2001); Liu et al. (2018a)
* Synnematospora *	Synnematous, macronematous	Ampulliform	Cylindrical to oblong; (1–)3-septate; catenate; brown	This study

#### ﻿ Neoberkleasmium micronesiacum

Taxon classificationFungiPleosporalesLentimurisporaceae

(Matsush.) J.Y. Zhang, K.D. Hyde & Y.Z. Lu comb. nov.

97CE7BF2-CE1F-5F1D-A8BC-999599D1FF10

Index Fungorum: IF904138

≡ Berkleasmium micronesiacum Matsush., Mycol. Mem. 2: 2 (1981)

##### Holotype.

USA, Guam, Mangilao, on the dead petiole of *Cocoris nuciferae*, 19 September 1980, Dried culture CMA, MFC-10321.

##### Description.

see Matsushima (1981).

##### Notes.

*Berkleasmium micronesiacum* was introduced by Matsushima (1981) based on morphological characteristics alone. However, phylogenetic analysis showed that *B. micronesiacum* and *B. nigroapicale* are separate from the monophyletic *Berkleasmium* lineage (Pinnoi et al. 2007; Liu et al. 2018a; Lu et al. 2018). Therefore, we transfer *Berkleasmium micronesiacum* and *B. nigroapicale* to *Neoberkleasmium*.

#### ﻿ Neoberkleasmium nigroapicale

Taxon classificationFungiPleosporalesLentimurisporaceae

(Bussaban, Lumyong, P. Lumyong, McKenzie & K.D. Hyde) J.Y. Zhang, K.D. Hyde & Y.Z. Lu comb. nov.

3BA74BBF-940B-5677-A272-11A74749879F

Index Fungorum: IF904139

≡ Berkleasmium nigroapicale Bussaban, Lumyong, P. Lumyong, McKenzie & K.D. Hyde, Fungal Diversity 8: 80 (2001)

##### Holotype.

THAILAND • Chiang Mai, Doi Suthep-Pui National Park, on dead pseudostems of *Amomum siamense* (*Zingiberaceae*), 15 October 2000, B. Bussaban CMUZS2 (POD 74415, holotype), ex-types living culture, BCC 8220 and HKUCC 7909.

##### Description.

see Bussaban et al. (2001).

##### Notes.

*Berkleasmium nigroapicale* was introduced by Bussaban et al. (2001), and this species was transferred to *Neoberkleasmium* based on phylogenetic evidence (Fig. 1).

#### ﻿ Synnematospora

Taxon classificationFungiPleosporalesLentimurisporaceae

J.Y. Zhang, Y.Z. Lu & K.D. Hyde gen. nov.

B8755F77-EB26-541B-AAA2-FC3D2C2072C6

Index Fungorum: IF904135

##### Etymology.

The genus name refers to the synnematous conidiophores.

##### Type species.

*Synnematospora pronephrii* J.Y. Zhang, Y.Z. Lu & K.D. Hyde.

##### Description.

Sexual morph: undetermined. Asexual morph: hyphomycetous. ***Colonies*** on natural substrate effuse, scattered, dark brown, with conidial masses at the upper part of conidiophores. ***Mycelium*** mostly immersed, composed of septate, hyaline to brown hyphae. ***Synnemata*** composed of compactly adpressed conidiophores, brown to dark brown. ***Conidiophores*** macronematous, synnematous, cylindrical, thick-walled, septate, brown. ***Conidiogenous cells*** blastic, discrete, terminal and lateral, ampulliform, flask-shaped, light brown. ***Conidia*** phragmosporous, solitary or catenate, cylindrical to oblong, straight or slightly curved, thick-walled, septate, constricted at septa, guttulate, brown. ***Conidial****secession* rhexolytic.

#### ﻿ Synnematospora pronephrii

Taxon classificationFungiPleosporalesLentimurisporaceae

J.Y. Zhang, Y.Z. Lu & K.D. Hyde sp. nov.

EA026ACC-E99D-527B-9C38-7A0935217AD6

Index Fungorum: IF904136

Fig. 5

##### Etymology.

The species epithet refers to the fungal host, “*Pronephrium penangianum*”.

**Figure 5.**
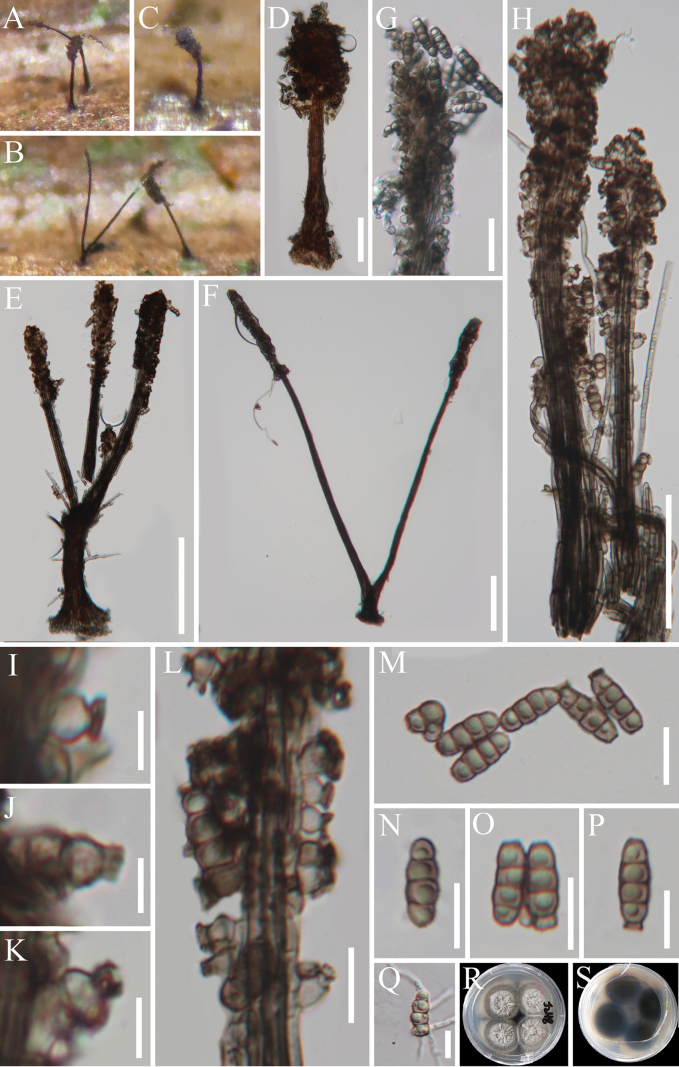
*Synnematospora pronephrii* (HAKS 129756, holotype). **A–C** Colonies on the host substrate; **D–H** Conidiophores; **I–L** Conidiogenous cells; **M–P** Conidia; **Q** Geminated conidium; **R, S** Pure culture from above and below. Scale bars: 100 μm (**D–F**); 20 μm (**G, L**); 50 μm (**H**); 5 μm (**I–K**); 10 μm (**M–Q**).

##### Diagnosis.

Differs from torula-like species in having macronematous, synnematous conidiophores.

##### Holotype.

HAKS 129756

##### Description.

Sexual morph: undetermined. Asexual morph: hyphomycetous. ***Colonies*** on natural substrate effuse, scattered, dark brown, with conidial masses at the upper half of conidiophores. ***Mycelium*** mostly immersed, composed of septate, smooth, hyaline to brown hyphae. ***Synnemata*** composed of compact, parallel, adpressed conidiophores, brown to dark brown, 328–564 μm long and up to 35 μm wide at the base. ***Conidiophores*** macronematous, synnematous, cylindrical, unbranched, thick-walled, septate, pale brown to brown, 1.8–2.7 µm wide. ***Conidiogenous cells***, blastic, discrete, ampulliform, flask-shaped, light brown, 2.5–5.3 × 2.8–4.2 µm (*x̄* = 4 × 3.4 µm, n = 20). ***Conidia*** phragmosporous, solitary or catenate, cylindrical to oblong with rounded or truncated ends, straight, thick-walled, (1–)3-septate when mature, constricted at septa, always with a single guttule in each cell, brown, 9–15 × 3.7–5 µm (*x̄* = 12.5 × 4.4 µm, n = 25). *Conidial secession* rhexolytic.

##### Culture characteristics.

Conidia germinating on WA within 15 h and germ tube produced from the ends of conidia. Colonies growing on PDA, reaching ca. 36 mm diameter in 20 days at 26 °C, circular, with entire margin, flat, with raise in the central part, dry, gray in the central part, brownness to pale brown towards the margin in front; dark brown in the center, paler to light brown towards the edge from below, and not producing pigmentation in cultures.

##### Material examined.

CHINA• Guizhou Province, Qianxinan Buyi and Miao Autonomous Prefecture, Anlong County, Xianheping National Forest Park, on dead stems of *Pronephrium penangianum* (*Thelypteridaceae*), 16 March 2022, J.Y. Zhang, J248 (HAKS 129756, holotype; GZAAS 23-0697, isotype), ex-type living culture KUNCC 23-13965.

##### Additional sequence.

ITS (PV862368).

##### Notes.

In the phylogenetic analysis, *Synnematospora pronephrii* formed a distinct and strongly supported lineage (81% ML/0.97 PP, Fig. 1), which is sister to *Bahusandhika indica* (GUFCC 18001) within *Lentimurisporaceae*. Members of *Bahusandhika* are torula-like (Pratibha et al. 2014; Crane and Miller 2016; Pem et al. 2024). Although the conidial characteristics of *Synnematospora pronephrii* resemble those of *Bahusandhika* species (Table 2), the species features macronematous, synnematous conidiophores. These characteristics also clearly distinguish it from other genera in *Lentimurisporaceae*, which possess sporodochial conidiomata (Liu et al. 2018a). Hence, we establish a new genus to accommodate *Synnematospora pronephrii* based on the morpho-phylogenetic evidence.

#### ﻿ Xenoberkleasmiaceae

Taxon classificationFungiPleosporalesXenoberkleasmiaceae

J.Y. Zhang, Y.Z. Lu & K.D. Hyde fam. nov.

BD9348A8-A9CA-5C31-AB0E-4C0C8EBD19AC

Index Fungorum: IF904140

##### Etymology.

The family name refers to the type genus.

##### Type genus.

*Xenoberkleasmium* N.G. Liu, Jian K. Liu & K.D. Hyde.

##### Description.

Sexual morph: Undetermined. Asexual morph: Hyphomycetous. ***Colonies*** on natural substratum effuse, scattered, punctiform or powdery, dark-brown to black, glistening. ***Mycelium*** partly immersed, partly superficial, composed of branched, septate, hyaline to pale brown hyphae. ***Conidiomata*** sporodochial. *Conidiophores* micronematous to macronematous, mononematous, sometime reduced to conidiogenous cells. ***Conidiogenous cells*** blastic, integrated, terminal, determinate. ***Conidia*** acrogenous, broadly ellipsoidal to obovoid, thick-walled, muriform, brown to olivaceous green, with or without guttules, usually with basal cell attached.

##### Notes.

*Xenoberkleasmiaceae* is introduced to accommodate the genus *Xenoberkleasmium*, which is characterized by sporodochial conidiomata and muriform, brown conidia. This group forms a distinct and well-supported clade within *Pleosporales* and shares a close relationship with the morphologically unrelated family *Hypsostromataceae*.

#### ﻿ Xenoberkleasmium

Taxon classificationFungiPleosporalesXenoberkleasmiaceae

N.G. Liu, Jian K. Liu & K.D. Hyde, Fungal Diversity 129:1–281 (2024)

BDCE9E3B-1728-55EE-A366-5D332C603FC7

##### Type species.

*Xenoberkleasmium chiangraiense* N.G. Liu, Jian K. Liu & K.D. Hyde 2024.

##### Notes.

*Xenoberkleasmium* was introduced as a monotypic genus to accommodate *X. chiangraiense* in *Pleosporales* (Liu et al. 2024a). In this study, *X. chiangraiense* clusters with *Berkleasmium crunisia*, *B. pandani*, and *B. typhae* in *Pleosporales*, distinct from the monophyletic lineage of *Berkleasmium*. Consequently, these three *Berkleasmium* species are transferred to *Xenoberkleasmium* based on shared morphological characteristics and congeneric phylogenetic placement.

#### ﻿ Xenoberkleasmium crinisium

Taxon classificationFungiPleosporalesXenoberkleasmiaceae

(Pinnoi) J.Y. Zhang, Y.Z. Lu & K.D. Hyde comb. nov.

A619E0E9-ED9E-5EE7-B624-8D3ADD7D9C2B

Index Fungorum: IF904141

≡ Berkleasmium crunisia Pinnoi, in Pinnoi, Jeewon, Sakayaroj, Hyde & Jones, Mycologia 99(3): 379 (2007)

##### Holotype.

THAILAND• Satun: Khuan Ka Long, on decaying rachis *Calamus* sp., 10 December 2004, A. Pinnoi in BIOTEC Bangkok Herbarium (BBH13084, holotype), ex-type culture, BCC 17023, 17024.

##### Description.

see Pinnoi et al. (2007).

##### Notes.

*Berkleasmium crunisia* was introduced by Pinnoi et al. (2007). In our phylogenetic analysis, *Berkleasmium crunisia* (BCC 17023) formed a distinct clade nested within *Xenoberkleasmium*. Morphologically, this species is characterized by sporodochial, punctiform colonies; macronematous, mononematous, clavate, aseptate, hyaline conidiophores; holoblastic conidiogenous cells; and muriform, oval to ellipsoidal, pale brown conidia (Pinnoi et al. 2007). These characteristics align with the generic concept of *Xenoberkleasmium*. Based on the molecular data and morphological characters, we therefore recognize *Berkleasmium crunisia* as a member of *Xenoberkleasmium*, proposing a new combination, *X. crinisium*.

#### ﻿ Xenoberkleasmium pandani

Taxon classificationFungiPleosporalesXenoberkleasmiaceae

(McKenzie) J.Y. Zhang, Y.Z. Lu & K.D. Hyde comb. nov.

6BC8F5DA-A481-5239-BDB2-031AABEA5C2A

Index Fungorum: IF904142

Fig. 6

≡ Berkleasmium pandani McKenzie, Mycotaxon 104: 24 (2008)

##### Holotype.

MALAYSIA • Genting Highlands, Ganung Buah, in the dead leaves of *Pandanus species* (*Pandanaceae*), 18 August 1992, E.H.C. McKenzie (PDD 60532).

**Figure 6.**
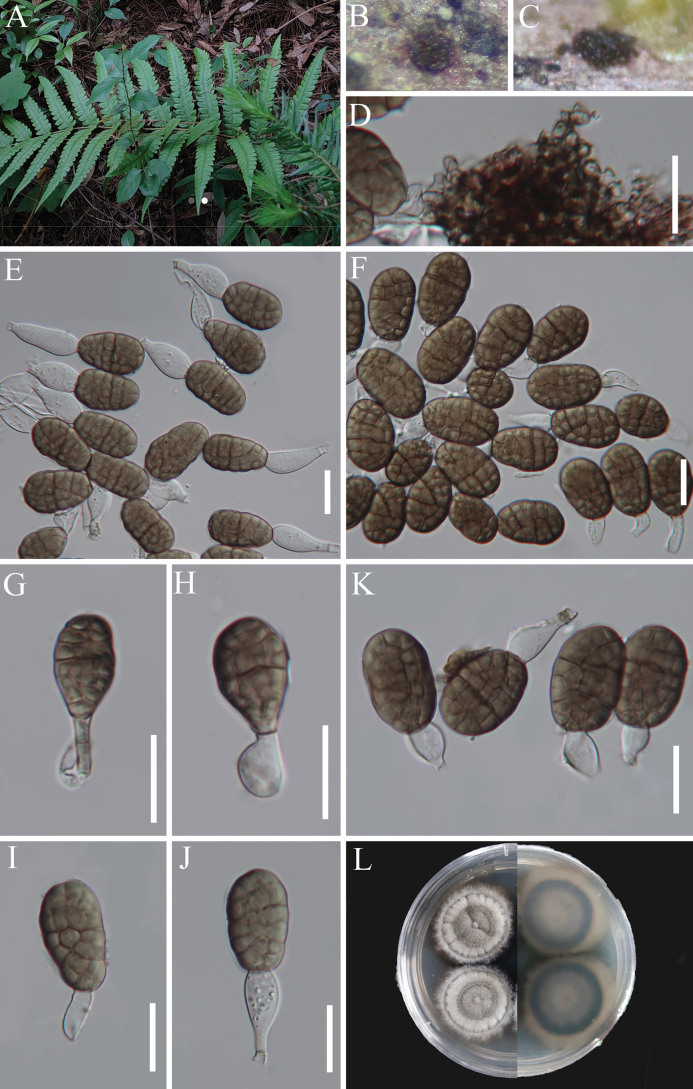
*Xenoberkleasmium pandani* (HKAS 147017). **A** The host; **B, C** Colonies on the host substrate; **D** Mycelium; **E–K** Conidiogenous cells and conidia; **L** Pure culture from above and below. Scale bars: 20 μm (**D–K**).

##### Description.

Sexual morph: Undetermined. Asexual morph: Hyphomycetous. ***Colonies*** on natural substrate effuse, superficial, scattered to gregarious, sporodochial, velvety, punctiform, brown to black. ***Mycelium*** mostly superficial, composed of branched, septate, hyaline to brown hyphae. ***Conidiophores*** macronematous, mononematous, cylindrical, hyaline to middle brown, up to 32 µm. ***Conidiogenous cells*** monoblastic, holoblastic, terminal, inverted lageniform, clavate, narrowed towards the base, truncate at apex after conidial secession, with dense cytoplasm, hyaline, 13–33 × (4–)8.5–12.8 µm (*x̄* = 23 × 10.5 µm, n = 20). ***Conidia*** 25–35 × 15–21 µm (*x̄* = 30 × 18 µm, n = 30), acrogenous, solitary, oval to ellipsoidal, usually broadly obtuse at apex and slightly truncate at base, thick-walled muriform, not constricted or slightly constricted at the septa, sometimes with a dark median septum, brown.

##### Culture characteristics.

Conidia germinating on WA within 15 h and germ tube produced from the ends of conidia. Colonies growing on PDA, reaching about. 42 mm diameter in one month at 26 °C, circular, with an indentation at the entire margin, flat, with a protuberance in the center, dry, celadon to gray from central part to margin from above; pale brown to celadon to brown from center towards the margin from below, and not producing pigmentation in cultures.

##### Material examined.

CHINA • Guizhou Province, Tongren City, Jiangkou County, (27°46'38"N, 108°45'22"E), on dead stems of *Woodwardia japonica* (*Blechnaceae*) in a forest near the roadside, 21 May 2022, J.Y. Zhang, F31-1 (HKAS 147017 = GZAAS 23-0824), living culture KUNCC 23-13876; • ibid., F32-1 (HKAS 129706 = GZAAS 23-0674), living culture, KUNCC 23-13878; CHINA • Guizhou Province, Qiandongnan Miao and Dong Autonomous Prefecture, Liping County, Yongcong Town, on dead fronds of *Blechnopsis orientalis* (*Blechnaceae*) in a forest, 27 March 2022, J.Y. Zhang, J352 (HKAS 147020), living culture KUNCC 23-14012.

##### Additional sequence.

KUNCC23-13876: ITS (PV862369); KUNCC 23-13878: ITS (PV862370); KUNCC 23-14012: ITS (PV862371).

##### Notes.

*Berkleasmium pandani* was introduced by McKenzie (2008), but sequence data was not provided. The morphological characters of our new collection align with the original diagnosis of the holotype (McKenzie 2008), including conidial sizes (25–35 × 15–21 µm vs. 27–34 × 18–22.5 µm). Although there are slight differences in the sizes of conidiogenous cells (13–33 × 8.5–12.8 µm vs. up to 45 µm), this may be attributed to differences in host and environmental conditions. Thus, we identified our new collections as *Berkleasmium pandani*. The molecular data for this species are generated for the first time. Phylogenetic analysis showed that our three new isolates form a distinct clade within *Xenoberkleasmium*. The characteristics of these new collections align with the generic concept of *Xenoberkleasmium*, and therefore, we have transferred them to *Xenoberkleasmium*.

Notably, Verma et al. (2019) introduced a new geographical record for *Berkleasmium pandani* based solely on morphological characteristics. However, based on the photographs and descriptions provided by Verma et al. (2019), we found that the elliptical to globose basal cells attached to the conidia were incorrectly described as conidiogenous cells. We therefore consider that the identification of the specimen (PAN 32722) as *Berkleasmium pandani* requires reevaluation, and we do not accept this specimen provided by Verma et al. (2019) as a valid record of *Berkleasmium pandani*.

#### ﻿ Xenoberkleasmium typhae

Taxon classificationFungiPleosporalesXenoberkleasmiaceae

(Somrith. & E.B.G. Jones) J.Y. Zhang, Y.Z. Lu & K.D. Hyde comb. nov.

EB2939D4-3181-5B3D-B2A3-B992E264E65E

Index Fungorum: IF904143

≡ Berkleasmium typhae Somrith. & E.B.G. Jones, Fungal Diversity 12: 170 (2003)

##### Holotype.

THAILAND • Pathum Thani, Klong Luang, in the decaying leaves of *Typha angustifolia*, August 2002, S. Somrithipol (SFC 1610 in BBH, holotype), ex-type living culture, BCC 12536 in BCC.

##### Description.

see Somrithipol and Jones (2003).

##### Notes.

*Berkleasmium typhae* was introduced by Somrithipol and Jones (2003) based solely on morphology. Pinnoi et al. (2007) provided sequence data for this species, demonstrating that *B. typhae* is closely related to *B. crunisia* (as *Xenoberkleasmium crinisium*). In our study, *B. typhae* clusters with three other *Xenoberkleasmium* species, prompting us to transfer it to *Xenoberkleasmium* as *X. typhae*. *Xenoberkleasmium typhae* matches well with the generic concept in having sporodochial, punctiform colonies and muriform, brown conidia (Pinnoi et al. 2007; Liu et al. 2024a).

###### ﻿*Pleosporales* genera incertae sedis

#### ﻿ Microlepicola

Taxon classificationFungiPleosporalesXenoberkleasmiaceae

J.Y. Zhang, Y.Z. Lu & K.D. Hyde gen. nov.

41BE7208-15F8-572D-B9C5-C9B26FC37E57

Index Fungorum: IF904145

##### Etymology.

The genus name refers to the fungal host “*Microlepia marginata*”.

##### Type species.

*Microlepicola guizhouensis* J.Y. Zhang, K.D. Hyde & Y.Z. Lu.

##### Description.

Sexual morph: ***Ascomata*** immersed, scattered, visible as dark spots that are slight raised. ***Ascomatal wall*** coriaceous, thin-walled, composed of brown cells of *textura angularis to textura prismatica* in surface view. ***Hamathecium*** filiform, pseudoparaphyses, hyaline. ***Asci*** 8-spored, bitunicate, cylindric-clavate, straight or slightly curved, with a short pedicel, apically rounded, with an ocular chamber. ***Ascospores*** overlapping, 2–3-seriate, fusiform, straight or slightly curved, 1-septate, not constricted at the septum, guttulate, surrounded by a thin and inconspicuous mucilaginous sheath. Asexual morph: Undetermined.

#### ﻿ Microlepicola guizhouensis

Taxon classificationFungiPleosporalesXenoberkleasmiaceae

J.Y. Zhang, K.D. Hyde & Y.Z. Lu sp. nov.

A6480669-F5F9-5063-96F2-4CDF9F3999B4

Index Fungorum: IF904146

Fig. 7

##### Etymology.

The species epithet refers to the collecting site, Guizhou province, China.

**Figure 7.**
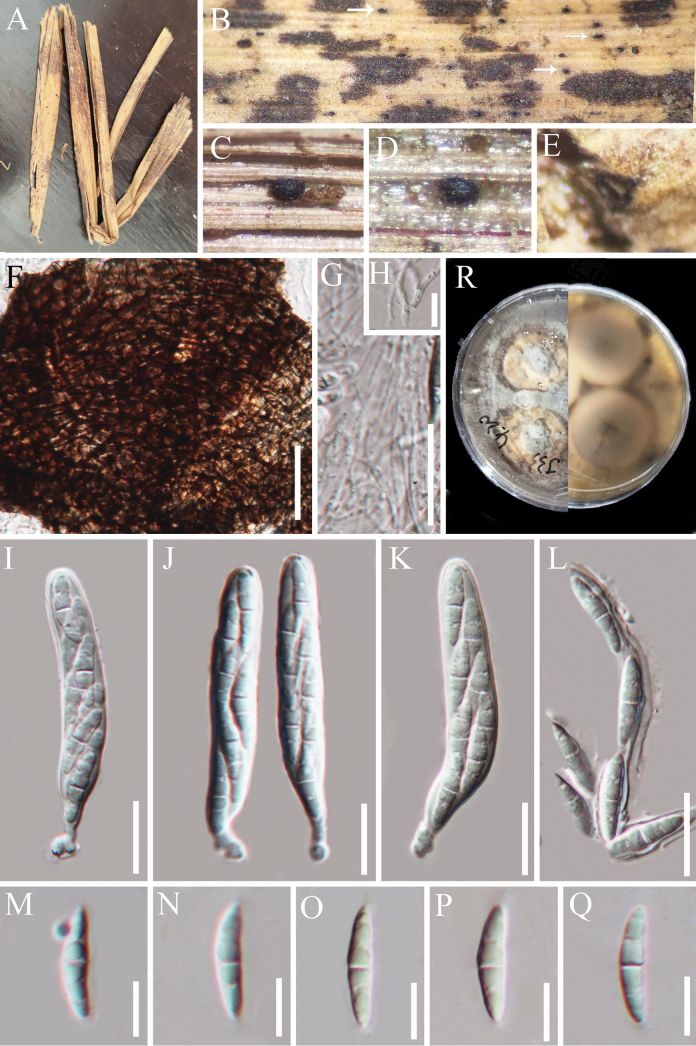
*Microlepicola guizhouensis* (HKAS 129777, holotype). **A** Host substrate; **B** Colonies on the host surface (arrows indicating ascomata); **C, D** Colonies from the lower side of the host epidermis; **E** Sectional view of the ascoma; **F** The surface of the ascoma; **G, H** Pseudoparaphyses; **I–K** Asci; **L–Q** Ascospores; **R** Pure culture. Scale bars: 30 μm (**F, G**); 10 µm (**H, M–Q**); 20 µm (**I–L**).

##### Diagnosis.

Differs from species in *Angustimassarina* and *Neobambusicola* in the shapes of hamathecium, asci and ascospores.

##### Holotype.

HKAS 129777.

##### Description.

Sexual morph: ***Ascomata*** deeply immersed, solitary or scattered, visible as dark spots that are slightly raised, which ascocarp passes through the thin host epidermis, exposing the mostly whole ascomata from lower side of the hosts. ***Ascomatal wall*** coriaceous, thin-walled, composed of brown cells of *textura angularis to textura prismatica* in surface view. ***Hamathecium*** filiform, remotely separate, pseudoparaphyses, hyaline, 1.3–2.6 µm wide. ***Asci*** 8-spored, bitunicate, cylindric-clavate, straight or slightly curved, with a short pedicel, apically rounded, with an ocular chamber, 68–85(–91) × 10.5–15.5 µm (*x̄* = 78 × 12.8 µm, n = 25). ***Ascospores*** overlapping, 2–3-seriate, fusiform, straight or slightly curved, 1-septate, not constricted at the septum, guttulate, 18.5–21.7 × 4–5.5 µm (*x̄* = 20 × 4.7 µm, n = 25), surrounded by a thin and inconspicuous mucilaginous sheath. Asexual morph: Undetermined.

##### Culture characteristics.

Ascospores germinating on WA within 13 h at 26 °C. Colonies on PDA, circular with entire margin, flat with raise in the center, dry, cotton, ashen in the center, khaki to grey towards the margin; light brown to brown-yellow from below, and not producing pigmentation in cultures.

##### Material examined.

CHINA • Guizhou Province, Qiandongnan Miao and Dong Autonomous Prefecture, Liping County, Yongcong Town, 26.0781942N, 109.1568557E, on dead fronds of *Microlepia marginata* (*Dennstaedtiaceae*) near a river in a forest, 27 March 2022, J.Y. Zhang, J333 (HKAS 129777, holotype; GZAAS 23-0715, isotype), ex-type living culture KUNCC 23-14007.

##### Additional sequence.

ITS (PV862372).

##### Notes.

In the phylogenetic analysis, our new isolate (KUNCC23-14007) forms a monophyletic clade in *Pleosporales*. *Microlepicola guizhouensis* exhibits some morphological similarities with several groups in the *Pleosporales*, such as *Angustimassarina* and *Neobambusicola*, particularly in the shapes of the hamathecium, asci, and ascospores (Thambugala et al. 2015; Mapook et al. 2020a; Yu et al. 2024). However, these groups are phylogenetically distinct and also differ in some morphological characteristics. *Microlepicola guizhouensis* differs from the two aforementioned genera in having immersed ascomata with clearly visible epidermal cell composition, ranging from textura angularis to textura prismatica. To avoid establishing a new family for a single species, *Microlepicola* was introduced as a new monotypic genus and assigned to *Pleosporales* genera incertae sedis.

#### ﻿ Xenopleopunctum

Taxon classificationFungiPleosporalesXenoberkleasmiaceae

J.Y. Zhang, Y.Z. Lu & K.D. Hyde gen. nov.

D76F2FC8-4543-581E-A265-7E3CA48805E2

Index Fungorum: IF904147

##### Etymology.

The genus name refers to the similar genus *Pleopunctum*.

##### Type species.

*Xenopleopunctum guizhouense* J.Y. Zhang, Y.Z. Lu & K.D. Hyde.

##### Description.

Sexual morph: Undetermined. Asexual morph: Hyphomycetous. ***Colonies*** on natural substrate effuse, superficial, scattered to gregarious, sporodochial, punctiform, brown to black. ***Mycelium*** partly immersed, partly superficial, composed of branched, septate, hyaline to brown hyphae. ***Conidiophores*** micro- to macronematous, mononematous, cylindrical, brown, often reduced to conidiogenous cells. ***Conidiogenous cells*** monoblastic, integrated, terminal, cylindrical, thick-walled, brown. ***Conidia*** acrogenous, mostly oval to ellipsoidal, muriform, constricted at septa, brown to dark brown, sometime slightly darked at the upper part, clearly darked at the septum, paler at the base, often with a cylindrical to subglobose, hyaline basal cell.

##### Notes.

*Xenopleopunctum* is introduced as a new genus to accommodate taxa exhibiting pleopunctum-like asexual morphology. *Xenopleopunctum* resembles *Pleopunctum* in having punctiform, brown colonies, monoblastic conidiogenous cells, and muriform, oval to ellipsoidal, dematiaceous conidia with a hyaline basal cell (Taylor and Hyde 2003; Liu et al. 2019; Yang et al. 2023). However, they are phylogenetically distinct, with *Pleopunctum* belonging to *Phaeoseptaceae* and *Xenopleopunctum* being sister to *Pseudomassarinaceae*, but not belonging to any existing family.

#### ﻿ Xenopleopunctum guizhouense

Taxon classificationFungiPleosporalesXenoberkleasmiaceae

J.Y. Zhang, Y.Z. Lu & K.D. Hyde sp. nov.

6EFA6695-7737-585E-BE59-F78EE502B0E7

Index Fungorum: IF904148

Fig. 8

##### Etymology.

The species epithet refers to its collecting site.

**Figure 8.**
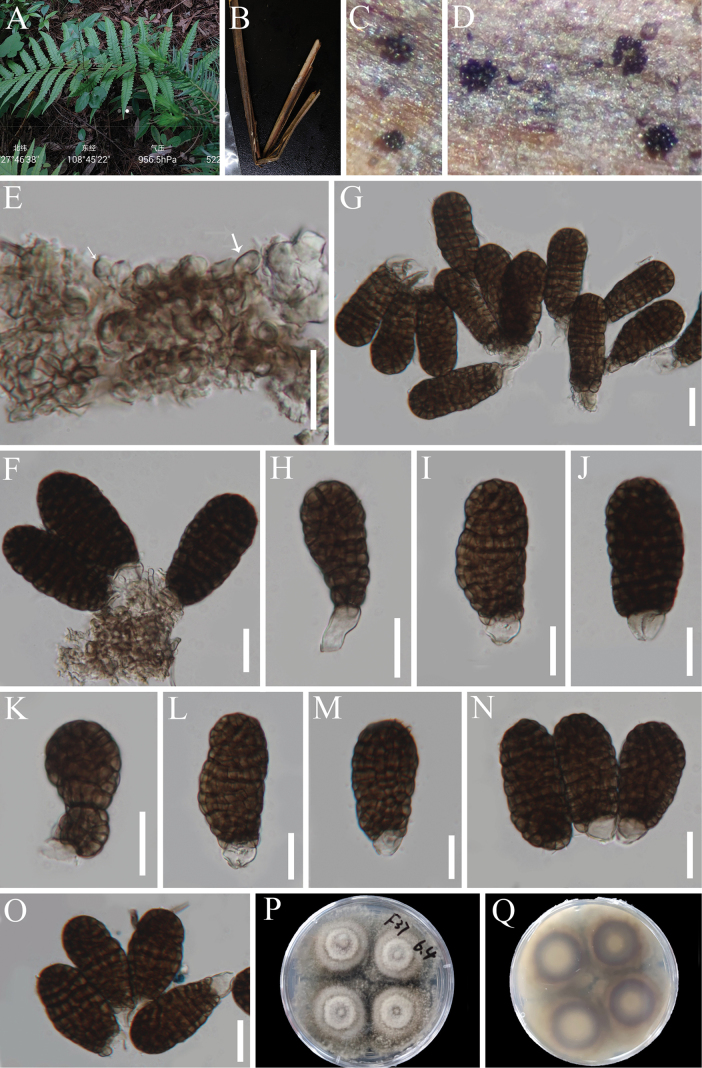
*Xenopleopunctum guizhouense* (HKAS 129707, holotype). **A** The host; **B** Host substrate; **C, D** Colonies on the host substrate; **E, F** Mycelium and conidiogenous cells (arrows indicating conidiogenous cells); **G–O** Conidia; **P, Q** Pure culture from above and below. Scale bars: 20 μm (**E–O**).

##### Diagnosis.

Differs from *X. sporodochiale* in having larger conidia (46.5–66.5 × 22–32.5 µm *vs.* 35–45(–47.8) × 18.3–25.5 µm) with diverse shapes.

##### Holotype.

HKAS 129707.

##### Description.

Sexual morph: Undetermined. Asexual morph: Hyphomycetous. ***Colonies*** on natural substrate superficial, effuse, scattered to gregarious, sporodochial, punctiform, brown to black. ***Mycelium*** mostly superficial, composed of branched, septate, hyaline to brown hyphae. ***Conidiophores*** micro- to macronematous, mononematous, cylindrical, brown, often reduced to conidiogenous cells. ***Conidiogenous cells*** monoblastic, integrated, terminal, cylindrical, thick-walled, brown, 4–6.5 µm (*x̄* = 5.1 µm, n = 20) wide. ***Conidia*** acrogenous, cylindrical to oval to irregular ellipsoidal, muriform, constricted at septa, brown to dark brown, sometime slightly darked at the upper part, 46.5–66.5 × 22–32.5 µm (*x̄* = 53.5 × 26.3 µm, n = 30), often with a cylindrical to subglobose, hyaline or subhyaline to pale brown basal cell, 8.5–14 × 6–15 µm.

##### Culture characteristics.

Conidia germinating on WA within 15 h and germ tube produced from the base of conidia. Colonies growing on PDA under natural light, reaching ca. 36 mm diameter after one month at 26 °C, circular with entire margin, flat with a protuberance in the center, dry, velvety, white to taupe brown in concentric circles from center towards margin in the front; pale brown in the center, followed by brown to pale brown in concentric circles towards the edge, not producing pigmentation in culture.

##### Material examined.

CHINA • Guizhou Province, Tongren City, Jiangkou County, 27°46'38"N, 108°45'22"E 522M, on dead petiole of *Woodwardia japonica*, in a distributed forest, 21 May 2022, J.Y. Zhang, F37 (HKAS 129707, holotype; GZAAS 23-0675, isotype), ex-type living culture KUNCC 23-13881; • ibid., F34 (HKAS 147018 = GZAAS 23-0825, paratype), living culture, KUNCC 23-13880; • ibid., F38-1 (HKAS 129708, paratype), living culture, KUNCC 23-13882.

##### Additional sequence.

KUNCC 23-13881: ITS (PV862373); KUNCC 23-13880: ITS (PV862374); KUNCC 23-13882: ITS (PV862375).

##### Notes.

Three collections representing a new species, *Xenopleopunctum guizhouense*, form an independent lineage that is sister to *X. sporodochiale* with strong support value (100% ML/1.00 PP, Fig. 1). *Xenopleopunctum guizhouense* and *X. sporodochiale* share a similar morphology in having sporodochial, punctiform colonies, integrated, monoblastic, brown conidiogenous cells, and muriform, brown conidia with a hyaline or subhyaline to pale brown basal cell. However, *Xenopleopunctum guizhouense* differs from *X. sporodochiale* in having larger conidia (46.5–66.5 × 22–32.5 µm vs. 35–45(–47.8) × 18.3–25.5 µm) with diverse shapes (cylindrical to oval to irregular ellipsoidal vs. oval to ellipsoidal). Additionally, a comparison of nucleotide base pairs of ITS, LSU, *RPB2*, SSU, and *tef1-α* between *X. guizhouense* (F37-KUNCC 23-13881) and *X. sporodochiale* (C22-HKAS 129694) shows 27/469 bp (6%, including 4 gaps), 12/857 bp (1%, with 2 gaps), 60/1062 bp (6%, 0 gaps), 2/1022 bp (-%, 0 gaps), and 50/908 bp (6%, with 1 gap) differences, confirming they are distinct species.

#### ﻿ Xenopleopunctum sporodochiale

Taxon classificationFungiPleosporalesXenoberkleasmiaceae

J.Y. Zhang, Y.Z. Lu & K.D. Hyde sp. nov.

89D34332-ED8B-5578-9974-1EFE25CC4495

Index Fungorum: IF904149

Fig. 9

##### Etymology.

The species epithet refers to its sporodochial conidiomata.

**Figure 9.**
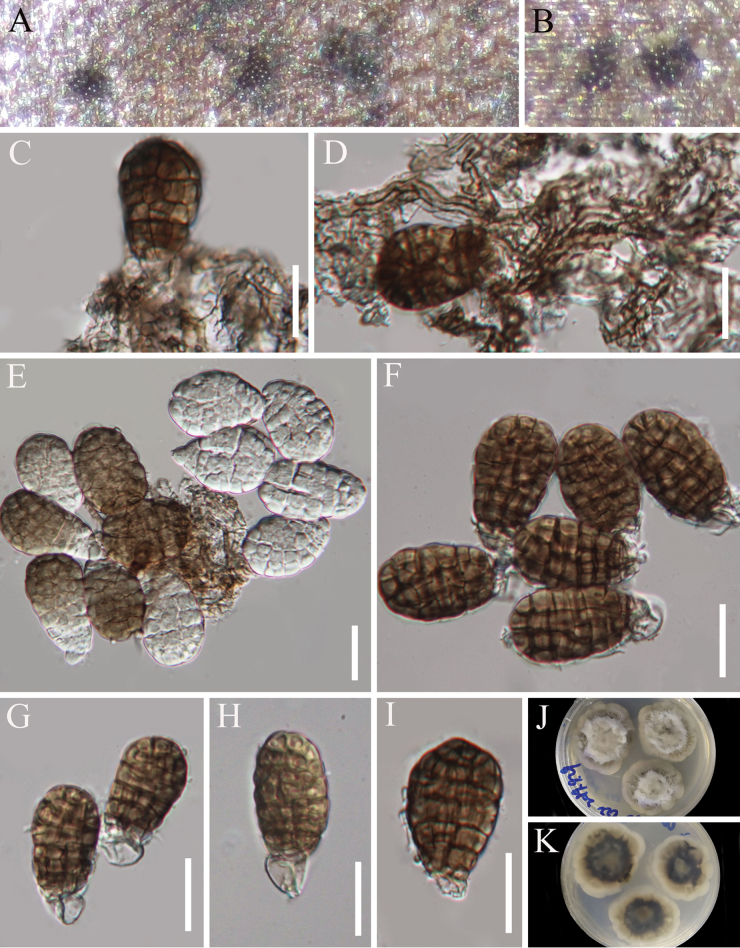
*Xenopleopunctum sporodochiale* (HKAS 129694, holotype). **A, B** Colonies on the host substrate; **C, D** Mycelium; **E–I** Conidia; **J, K** Pure culture from above and below. Scale bars: 20 μm (**C–I**).

##### Diagnosis.

Differs from *Xenopleopunctum guizhouense* in having small conidia (35–45(–47.8) × 18.3–25.5 µm *vs.* 46.5–66.5 × 22–32.5 µm).

##### Holotype.

HKAS 129694

##### Description.

Sexual morph: Undetermined. Asexual morph: Hyphomycetous. ***Colonies*** on natural substrate effuse, superficial, scattered to gregarious, sporodochial, punctiform, brown to black. ***Mycelium*** partly superficial, partly immersed, composed of branched, septate, hyaline to brown hyphae. ***Conidiophores*** micronematous, mononematous, reduced to conidiogenous cells. ***Conidiogenous cells*** monoblastic, integrated, terminal, cylindrical, thick-walled, brown, 3–6.2 × 2.5–3.1 µm (*x̄* = 4.6 × 2.8 µm, n = 15). ***Conidia*** acrogenous, oval to ellipsoidal, muriform, slightly constricted at septa, hyaline or subhyaline when immature, hyaline to brown from base to upper part during maturation, brown when mature, darked at the septum, 35–45(–47.8) × 18.3–25.5 µm (*x̄* = 40.7 × 21.5 µm, n = 30), often with a cylindrical to subglobose, hyaline or subhyaline to pale brown basal cell, 7.5–12 × 7.3–12 µm (*x̄* = 9.8 × 10.9 µm, n = 20).

##### Culture characteristics.

Conidia germinating on WA within 13 h and germ tube produced from the base of conidia. Colonies growing on PDA under natural light, reaching ca. 26 mm diameter after one month at 26 °C, irregular or subrotund in shape with lobate edge, raised with convex in the center, dry, fluff, white to pale brown towards the edge from the front; middle brown to dark brown to white from the center to margin from below, and not producing pigmentation in culture.

##### Material examined.

CHINA • Guizhou Province, Zunyi City, Chishui County, Hushi Town, Chishui Alsophila Natural Reserve, on dead frond stalks of *Cyatheaceae* sp., 22 September 2019, J.Y. Zhang, C22 (HKAS 129694, holotype, GZAAS 23-0774, isotype), living culture, GZCC 23-0742.

##### Additional sequence.

ITS (PV862376).

##### Notes.

*Xenopleopunctum sporodochiale* formed a distinct clade and shared a sister relationship with *X. guizhouense*, which confirmed they are separate species. The difference between these two species was mentioned above.

## ﻿Discussion

China is presently the second richest source of fungi associated with ferns and their allies (Bao et al. 2025; Zhang et al. 2025). In this study conducted in Guizhou Province, China, we isolated 14 strains from ferns identified in *Dothideomycetes*. Our phylogenetic analysis based on LSU, *RPB2*, SSU, and *tef1-α* sequence data supports a natural classification, leading to scientific adjustments in some lineages (Phukhamsakda et al. 2022). We propose one new family, six new genera, six new species, and five new combinations. This discovery further supports the previous study by Zhang et al. (2025) regarding the diversity and prevalence of *Dothideomycetes* within fungal communities associated with fern hosts. These findings enhance our understanding of fern-related fungal diversity in China and lay the groundwork for future studies on the ecological roles of fungi linked to ferns and their allies.

*Berkleasmium* (Zobel 1854) has traditionally been considered polyphyletic and phylogenetically unresolved, as its broadly delimited concept accommodates sporodochial species that cluster in different families (Pinnoi et al. 2007; Hyde et al. 2016; Lu et al. 2017; Tibpromma et al. 2017). Tanney and Miller (2017) provided the asexual–sexual morph connection for *B. concinnum* and placed this generic type of *B. concinnum* in *Tubeufiaceae*, *Tubeufiales*, based on phylogenetic analysis. Subsequently, Lu et al. (2018) accepted only seven species in *Berkleasmium* sensu stricto based on strong phylogenetic evidence and similar sexual morph characteristics (Tanney and Miller 2017; Lu et al. 2018). *Berkleasmium* now has two asexual morph types of helicosporous and dictyosporous hyphomycetes, represented by *B. concinnum*. In this study, three *Berkleasmium* species (*B. crunisia*, *B. pandani*, and *B. typhae*) are transferred to *Xenoberkleasmium*, which shares similar morphological characteristics with the type species *X. chiangraiense* in having punctiform colonies; macronematous, mononematous conidiophores; monoblastic, cylindrical to inverted lageniform, hyaline or pale brown conidiogenous cells; and muriform, oval to ellipsoidal, black conidia with oblique and irregular septa (Liu et al. 2024a). The sporodochial dictyoconidia of *Berkleasmium* are characterized by cylindrical, brown conidiogenous cells and conidia with regular horizontal septa, which can be differentiated from *Xenoberkleasmium* (Hughes 1958; Bussaban et al. 2001; Tanney and Miller 2017; Verma et al. 2019).

Similarly, two species of *Berkleasmium* (*B. micronesiacum* and *B. nigroapicale*) form a phylogenetically monophyletic clade within the morphologically related family *Lentimurisporaceae*, with sporodochial conidiomata (Liu et al. 2018a). Thus, a new genus, *Neoberkleasmium*, is proposed to accommodate two new combinations, *N. micronesiacum* and *N. nigroapicale*. Furthermore, as many *Berkleasmium* species lack molecular data, they were not accepted by Lu et al. (2018), rendering their generic position dubious and necessitating clarification with new collections and isolates (Bussaban et al. 2001; Somrithipol and Jones 2003; Zhao and Zhang 2004; Qu et al. 2014; Tibpromma et al. 2017).

The new genus *Xenopleopunctum* morphologically resembles *Pleopunctum (Phaeoseptaceae)*, exhibiting an asexual morphology characterized by punctiform, brown colonies; monoblastic conidiogenous cells; and muriform, oval to ellipsoidal, dematiaceous conidia with a hyaline basal cell (Liu et al. 2019; Boonmee et al. 2021; Xu et al. 2023b; Yang et al. 2023). Phylogenetically, *Xenopleopunctum* shares a sister relationship with *Pseudomassarina*, which was placed within *Pseudomassarinaceae*. *Pseudomassarina clematidis* is the only accepted species in this family and exhibits similar sexual morphological traits to *Lignosphaeria*, a genus belonging to *Phaeoseptaceae* (Thambugala et al. 2015; Pem et al. 2024). Both genera possess immersed to erumpent, coriaceous ascomata with a central ostiole; cylindrical to clavate asci with an apically rounded and ocular chamber; and hyaline, septate, fusiform conidia (Thambugala et al. 2015; Phukhamsakda et al. 2020; Pem et al. 2024). Given these similarities, it is possible that our new genus, *Xenopleopunctum*, may represent an asexual morph within *Pseudomassarinaceae*, potentially related to *Pseudomassarina*. While we were unable to culture the sexual morph of *Xenopleopunctum*, this prevents us from verifying our hypothesis. Therefore, more collections and analyses are necessary to confirm any taxonomic assumptions and establish a connection to the sexual morph of *Xenopleopunctum*.

## Supplementary Material

XML Treatment for Muyocopronales

XML Treatment for Cyatheomyces

XML Treatment for Cyatheomyces synnematosus

XML Treatment for Pseudopalawaniella

XML Treatment for Pseudopalawaniella woodwardiae

XML Treatment for Pleosporales

XML Treatment for Neoberkleasmium

XML Treatment for Neoberkleasmium micronesiacum

XML Treatment for Neoberkleasmium nigroapicale

XML Treatment for Synnematospora

XML Treatment for Synnematospora pronephrii

XML Treatment for Xenoberkleasmiaceae

XML Treatment for Xenoberkleasmium

XML Treatment for Xenoberkleasmium crinisium

XML Treatment for Xenoberkleasmium pandani

XML Treatment for Xenoberkleasmium typhae

XML Treatment for Microlepicola

XML Treatment for Microlepicola guizhouensis

XML Treatment for Xenopleopunctum

XML Treatment for Xenopleopunctum guizhouense

XML Treatment for Xenopleopunctum sporodochiale

## ﻿Additional information

### Use of AI

No use of AI was reported.

### Adherence to national and international regulations

All the fungal strains used in this study have been legally obtained, respecting the Convention on Biological Diversity (Rio Convention).

### Author ORCIDs

Jing-Yi Zhang https://orcid.org/0000-0003-0606-6169

Kevin D. Hyde https://orcid.org/0000-0002-2191-0762

Ming-Fei Yang https://orcid.org/0000-0002-5414-921X

Ya-Ru Sun https://orcid.org/0000-0001-5549-1028

Xing-Juan Xiao https://orcid.org/0009-0003-8769-4534

Ze-Bin Meng https://orcid.org/0000-0002-9995-6605

Dan-Feng Bao https://orcid.org/0000-0002-5697-4280

Yong-Zhong Lu https://orcid.org/0000-0002-1033-5782

### Data availability

All of the data that support the findings of this study are available in the main text or Supplementary Information.

## Supplementary materials

Supplementary material 1 Tree alignment Data type zip Explanation note The obtained alignment was used for phylogenetic study. https://binary.pensoft.net/file/1494391This dataset is made available under the Open Database License (http://opendatacommons.org/licenses/odbl/1.0/). The Open Database License (ODbL) is a license agreement intended to allow users to freely share, modify, and use this Dataset while maintaining this same freedom for others, provided that the original source and author(s) are credited.Jing-Yi Zhang, Kevin D. Hyde, Ming-Fei Yang, Ya-Ru Sun, Xing-Juan Xiao, Ze-Bin Meng, Dan-Feng Bao, Yong-Zhong Lu
